# Assessing benthic invertebrate vulnerability to ocean acidification and de-oxygenation in California: The importance of effective oceanographic monitoring networks

**DOI:** 10.1371/journal.pone.0317906

**Published:** 2025-02-18

**Authors:** Meghan Zulian, Esther G. Kennedy, Sara L. Hamilton, Tessa M. Hill, Genece V. Grisby, Aurora M. Ricart, Eric Sanford, Ana K. Spalding, Manuel Delgado, Melissa Ward

**Affiliations:** 1 Bodega Marine Laboratory, Department of Earth & Planetary Sciences, University of California Davis, Davis, California, United States of America; 2 Oregon Kelp Alliance, Port Orford, Oregon, United States of America; 3 Institut de Ciències del Mar, Consejo Superior de Investigaciones Científicas (ICM-CSIC), Barcelona, Spain; 4 Bodega Marine Laboratory, Department of Ecology and Evolutionary Biology, University of California Davis, Davis, California, United States of America; 5 School of Public Policy, Oregon State University, Corvallis, Oregon, United States of America; 6 Smithsonian Tropical Research Institute, Ancon, Panama, Republic of Panama; 7 Department of Geography, San Diego State University, San Diego, California, United States of America; Central Marine Fisheries Research Institute, INDIA

## Abstract

Greenhouse gas emissions from land-use change, fossil fuel, agriculture, transportation, and electricity sectors expose marine ecosystems to overlapping environmental stressors. Existing climate vulnerability assessment methods analyze the frequency of extreme conditions but often minimally consider how environmental data gaps hinder assessments. Here, we show an approach that assesses vulnerability and the uncertainty introduced by monitoring data gaps, using a case study of ocean acidification and deoxygenation in coastal California. We employ 5 million publicly available oceanographic observations and existing studies on species responses to low pH, low oxygen conditions to calculate vulnerability for six ecologically and economically valuable benthic invertebrate species: red sea urchin (*Mesocentrotus franciscanus*), purple sea urchin (*Strongylocentrotus purpurpatus*), warty sea cucumber (*Apostichopus parvimensis*), pink shrimp (*Pandalus jordani*), California spiny lobster (*Panulirus interruptus*), and Dungeness crab (*Metacarncinus magister*). Further, we evaluate the efficacy of current monitoring programs by examining how data gaps heighten associated uncertainty. We find that most organisms experience low oxygen (<35% saturation) conditions less frequently than low pH ( < 7.6) conditions. It is only deeper dwelling (>75 m depth) life stages such as Dungeness crab adults and pink shrimp embryos, juveniles, and adults that experience more frequent exposure to low oxygen conditions. Adult Dungeness crabs experience the strongest seasonal variation in exposure. Though these trends are intriguing, exposure remains low for most species despite well-documented pH and oxygen declines and strengthening upwelling in the central portions of the California Current. Seasonal biases of data collection and sparse observations near the benthos and at depths where organisms most frequently experience stressful conditions undermine exposure estimates. Herein we provide concrete examples of how pink shrimp and Dungeness crab fisheries may be impacted by our findings, and provide suggestions for incorporating oceanographic data into management plans. By limiting our scope to California waters and assessing the limitations presented by current monitoring coverage, this study aims to provide a granular, actionable framework that policymakers and managers can build from to prioritize targeted enhancements and sustained funding of oceanographic monitoring recommendations.

## 1. Introduction

Natural biogeochemical processes, carbon emissions, and agricultural activities create pervasive low pH and low oxygen conditions in many coastal marine environments [e.g., 1–6]. In areas of high primary productivity, decomposing phytoplankton blooms fed by agricultural runoff or natural nutrient loading processes (i.e., upwelling) consume seawater oxygen and reduce pH [e.g., 5,7]. Regional acidic, hypoxic conditions have reached new extremes as global carbon emissions lower seawater pH [e.g., 8–10] and warm surface waters, reducing mixing and oxygen transfer to deeper waters [e.g., 3,11,12]. These processes contribute substantially to localized extreme conditions [e.g., 1,3,13], and global trends of ocean acidification and deoxygenation that increasingly threaten coastal ecosystems, reliant communities, and industries [e.g., 13–16].

California’s comprehensive ocean monitoring network and oceanographic regime as an upwelling system make it an excellent ‘natural laboratory’ for examining the impacts of ocean acidification and deoxygenation. The California Current System (CCS) experiences strong seasonal lows and long-term declines in dissolved oxygen (DO) and pH [17–21]. Intense alongshore winds transporting surface waters offshore are replaced with deeper, low pH, low oxygen waters [e.g., 22–24]. In summer, winds subside, and increasingly warm surface waters limit oxygen transfer to deeper waters [stratification; e.g., 3,11,12]. Warming temperatures may amplify these seasonal processes. There is evidence for stronger alongshore winds in recent years, importing acidic, oxygen-poor waters to shallower depths [e.g., 24–27]. Long-term monitoring in the central and southern CCS shows that below 100 m, seawater DO declined by 40% in the last two decades, and pH is declining at nearly twice the global average rate [e.g., 19,20]. Regional Ocean Modeling System (ROMS) projections predict continued, rapid declines in DO and pH in the coming decades [28–32].

Projected ocean acidification and deoxygenation in the CCS could harm ecologically and economically important species of decapods and echinoderms at every life stage [e.g., 33–35]. Evidence from laboratory experiments suggests that decapod species from the CCS could experience various harmful effects, including delayed embryo hatching, exoskeleton dissolution, mechanoreceptor damage in larvae; increased respiration, reduced hemolymph pH, impaired olfactory pathways that alter foraging behavior in adults, and lower exposure at all life stages under sufficiently extreme, and prolonged conditions [e.g., 30,35–39]. Likewise, echinoderm species found in the CCS may experience reduced growth and increased respiration in larvae and reduced fluid pH and grazing rates in adults [e.g., 31,40–42], though there is robust evidence that some populations of echinoderms may tolerate low pH and low oxygen conditions, conferred by acclimatization and adaptation to local stress history, namely strong upwelling regimes [e.g., 43–54].

Predicting organisms’ vulnerability to ocean acidification and deoxygenation is incomplete without understanding their exposure to extreme conditions. Vulnerability is defined here as “the degree to which a system is susceptible to and is unable to cope with adverse effects” [55]. Prior studies modeled current and future exposure and vulnerability (i.e., Regional Ocean Modeling Systems (ROMS); GFDL ESM2M earth system model; Intergovernmental Panel on Climate Change CO_2_ scenarios, and Biogeochemical Element Cycling) to assess the benthic invertebrates’ exposure and vulnerability to low oxygen, low pH conditions in the CCS [i.e., 28,30–32,56]. ROMS models have extensive spatial coverage and are highly effective at translating individual impacts to population vulnerability. However, their resolution makes these models less accurate for nearshore conditions, and researchers’ choice to use maximum exposure and focus on end-of-century projections limits use for regional resource management [e.g., 57,58].

Direct oceanographic observations accurately estimate nearshore exposure and vulnerability, and are favored by resource managers over model outputs [e.g., 57,58]. Year-round fixed-location observations provide high temporal resolution at the expense of spatial coverage, while oceanographic surveys are spatially extensive but temporally limited [e.g., 59,60]. Individually, monitoring programs are insufficient for constraining exposure and vulnerability as they do not cover the full range of conditions experienced by organisms through space and time. However, a recent extensive compilation of oceanographic monitoring data across the CCS [61] makes it possible to attempt a vulnerability analysis using direct oceanographic observations.

To inform effective coastal policy, we need to understand species vulnerability to ocean acidification and deoxygenation, and our degree of certainty in that vulnerability. The “Managing for Climate Change” section of the 2018 California Department of Fish and Wildlife Master Plan for Fisheries explicitly calls for researchers and managers to develop and evaluate tools for identifying vulnerable species, as this information would inform many critical facets of management including the development of enhanced status reports (ESR) and fisheries management plans (FMP). The Master Plan also states that FMPs should explicitly incorporate information on changes to species’ life histories, such as anticipated alterations to feeding, growth, and other life history patterns, and any potential impacts of ocean chemistry. Here we combine standard methods in ecological vulnerability assessments [i.e., 28,30,62,63], with monitoring gaps analyses [i.e., 64] to investigate: 1) how vulnerable decapod and echinoderm species are to ocean acidification and deoxygenation in the CCS and 2) how well oceanographic monitoring networks capture exposure to acidic, hypoxic conditions. By making use of compiled oceanographic data and assessing the spatiotemporal bias of our monitoring networks, we ask whether exposure and vulnerability to ocean acidification and deoxygenation in the last decade varies among a set of key species, what monitoring gaps create high uncertainty in these calculations, and in turn, what additional monitoring could improve our understanding of their exposure and vulnerability?

## 2. Methods

We selected six invertebrate species for this study: red sea urchin (*Mesocentrotus franciscanus*), purple sea urchin (*Strongylocentrotus purpurpatus*), warty sea cucumber (*Apostichopus parvimensis*), pink shrimp (*Pandalus jordani*), California spiny lobster (*Panulirus interruptus*), and Dungeness crab (*Metacarnicus magister*). Beyond their ecological and economic importance, we chose a suite of species that represent various biogeographic ranges, larval seasonalities and durations, and juvenile and adult depth distributions. We do not aim to provide comprehensive ocean acidification and deoxygenation vulnerabilities for coastal assessment benthic invertebrates [i.e., 56], but rather provide illustrative examples of how existing literature and oceanographic monitoring networks limit our vulnerability estimates in organisms of differing life histories and distributions.

**Fig 1:**
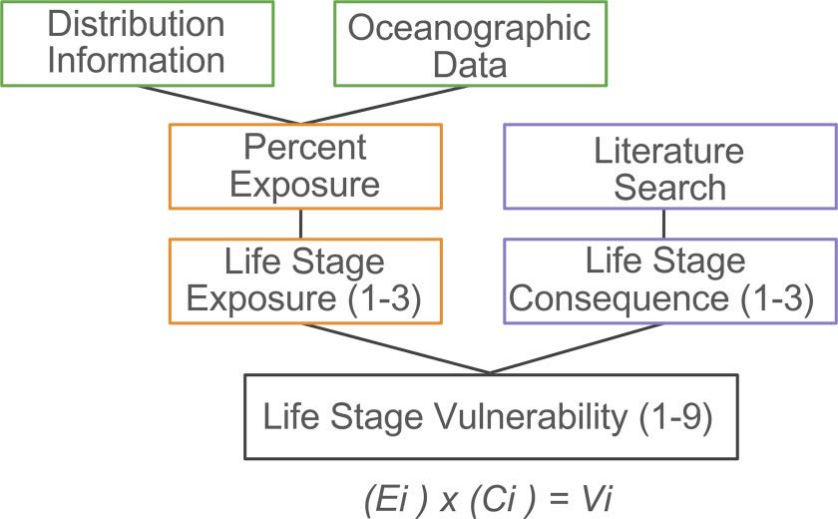
Schematic of the vulnerability assessment process and components [adapted from^28^,^30^]. Exposure score for each life stage (E_i_) is multiplied by consequence for each life stage (C_i_) to produce life stage vulnerabilities (V_i_). Information on species distribution throughout life history sets limits for what oceanographic data to include in exposure calculations. Numbers in parentheses represent the range of exposure, consequence, and vulnerability - the product of exposure and consequence scores.

To assess these organisms’ vulnerability to ocean acidification and deoxygenation, we investigated the overlap of stressful oxygen and pH conditions with their distribution throughout life history. Using a semi-quantitative approach developed by Hodgson et al., [28] we calculated life stage vulnerability (V_pH_ and V_DO_) as the product of exposure to low pH and oxygen (E_pH_ and E_DO_) and corresponding consequences (C_pH_ and C_DO_), each having a score from 1–3 (Fig 1). We assume population vulnerability to individual stressors is equal to the most vulnerable stage, which may be an overestimate [28,30].

To assess current monitoring networks’ capacity for detecting organisms’ exposure to chemical stressors, we also investigated the overlap of oxygen and pH monitoring with the distribution of the six coastal invertebrates throughout their life history. The degree of overlap, and our certainty in species distributions throughout life history, are reflected in the uncertainty to exposure (U_epH_ and U_eDO_), scored from 1–3.

### 2.1. Biogeographic distributions

**Fig 2:**
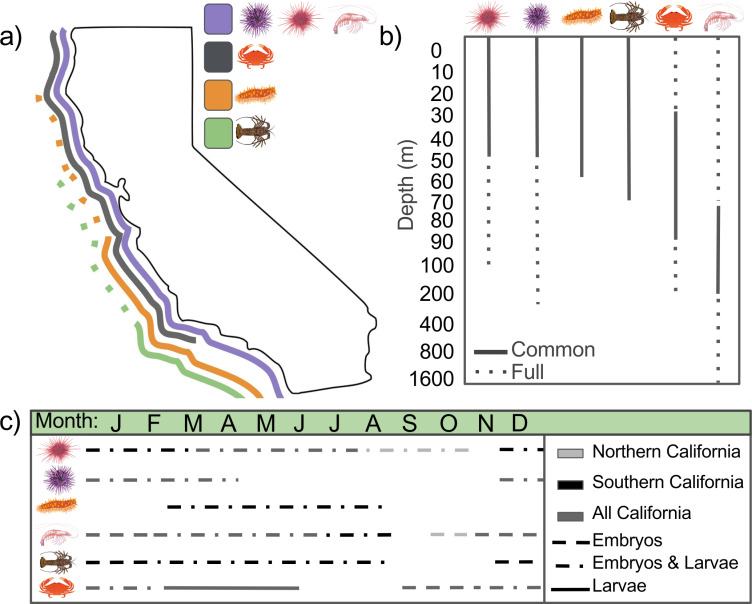
Distributions of study species throughout life history. A map of biogeographic distributions (a) and a schematic of adult depth distributions (b), with solid lines representing the common range and dotted lines representing rare occurrences. Also, a schematic of early life history seasonality (c), with the presence of embryo development represented by a dashed line, concurrent spawning, embryo, and larval development represented by dashed and dotted lines, and larval development represented by a solid line. Lines are colored by presence across their biogeographic distribution, with light gray for just northern California ( > 37.7°N), black for just southern California (<37.7°N), and dark gray for both.

To set the bounds for exposure calculations, we used a literature review and consulted with experts on the biogeography, seasonality of spawning, larval development, and juvenile and adult depth range of each species (Table 1 in S1 File; Fig 2). The amount of detailed information on life stage and species distributions strongly depends on their economic value and the type of fishing equipment used in their extraction. Though we employ the best available data, there are often few studies that characterize species ontogeny and distributions, creating significant uncertainty which we address further in our discussion.

### 2.2. Consequences and corresponding thresholds

We define consequence as “the degree to which a life stage demonstrates negative responses to a particular stressor” [28]. We estimated consequences for each life stage from each species based on responses from laboratory experiments or field observations reported in the literature (n = 55 studies). However, such studies do not exist for some species, forcing us to draw on research on species in the same genus or suborder (Tables 2 and 3 in S1 File ). Studies report numerous responses to low pH, low oxygen conditions: mortality, delayed hatching, slower growth, development, and feeding rates, and sometimes no response (Tables 2 and 3 in S1 File). We assigned lethal responses a consequence score of 3, sublethal responses a consequence score of 2, and no response or demonstrated resilience a consequence score of 1 (Tables 2 and 3 in S1 File ).

We also used pH and oxygen conditions reported in the literature review to calculate physiologically relevant DO and pH thresholds (n = 43, Tables 1 and 2 in S2 File). Before doing so, we had to convert all study conditions into similar units. Most acidification studies reported pH, so generally no conversions were needed. However, a few studies (n = 2) only reported *p*CO2, with no other carbonate *p*arameters, so we were unable to calculate pH and include their treatment conditions into our threshold calculations. By contrast, studies examining the effects of hypoxic conditions reported DO concentrations in many different units, which we converted to percent saturation (% sat) using reported DO values, temperatures, and salinities [*respR* package; 65]. For some studies that reported DO and temperature but not salinity (n = 5), we used the publicly available MOCHA dataset [61] to calculate the salinities of marine laboratory intake waters during experiments [42,66,67] and for field studies that correlated die-off events with low oxygen conditions [2,68]. If the salinity range was narrow ( < 2 PSU), we used the average to convert oxygen values to % sat. In developing thresholds, we did not include studies outside the U.S. West Coast that did not report salinities (n = 2). We also did not include thresholds from experiments that co-varied multiple environmental parameters, and did not have treatments that isolated the effects of individual stressors (n = 3).

Using pH and DO conditions from the literature, we calculated pH and DO averages that could represent physiologically relevant thresholds across all species (n = 43, Tables 1 and 2 in S2 File). In developing our main thresholds, we first averaged the treatment conditions from each study, to ensure multiple treatments reported by one study did not outweigh an average treatment reported by another. We then averaged by species, so as to not overrepresent highly studies species. Using the study, and species averaged treatment values, we took one final average of treatment conditions used on CCS species. We chose this approach for our main thresholds to ensure 1) we incorporate more data than is available just for study species, and 2) the thresholds reflect responses from organisms experiencing similar oceanographic conditions (i.e., upwelling). Finally, we rounded resulting pH thresholds to the nearest 0.05 pH units to reflect the limited accuracy of glass electrode systems used in some cited studies [66], and rounded saturation to the nearest 1%, so as to not overstate the precision of conversions made using estimated salinities [2,42,67–69]. Our resulting thresholds lie at pH = 7.6 and DO = 33%. To test the sensitivity of our vulnerability scores to these thresholds, we also examined % exposure and corresponding exposure scores thresholds that reflect the average values evoking lethal responses across all studies (24% sat, pH = 7.55; Tables 1 and 2 in S2 File), and the average treatment values experienced by species included in this study (39% sat, pH = 7.65; Tables 1 and 2 in S2 File).

We recognize that our approach to developing consequences and thresholds is limited. It does not take into account the different stress history and adaptation of coastal populations that exist along the mosaic of upwelling conditions throughout California [i.e., 18,70–72]. Ideally, we would incorporate such geographically-mediated sensitivities and histories into our analysis; however, this information is only available for larval red and purple urchin, and juvenile red urchin [e.g., 43–54,72,73]. By not taking into account the role of local stress history, we likely overestimate the consequences experienced by some populations of species, while underestimating others. On a separate note, thresholds are not based on species-specific responses to treatment conditions, which we also ideally use. We chose a universal threshold reflecting sensitivities of CCS species, as we felt this was more appropriate than using sensitivities of organisms that are adapted to vastly different oceanographic conditions, as used in developing consequence scores. Further, any uncertainty in exposure scores associated with a species-specific threshold would become merged with uncertainty analyses (e.g., quality of oceanographic data coverage; knowledge of life history, depth, and biogeographic distributions), complicating interpretations of *E*_*i*_ alongside *Ue*_*i*_. The use of universal thresholds also eases comparison with other CCS vulnerability assessments [28,30], which would not be possible with species and life stage-specific thresholds.

### 2.3. Exposures

Using information on species distribution (latitude, depth, distance offshore) and oceanographic data, we calculated exposure using all pH and oxygen data from within the bounds set for each organism’s life stages. Exposure here is defined as: the duration of weeks or months that organisms are exposed to average conditions that fall below thresholds that we anticipate will evoke negative physiological responses. Our exposure calculations use the MOCHA dataset, a synthesis of 13.7 million oceanographic data throughout the U.S. continental West Coast [61]. We also used these measured data to calculate an additional 950,298 data points from other carbonate parameters [*Seacarb* package in R; 74]. We limited our scope to data within California, US (32.5°N to 42°N) collected from 2010 - 2021 to ensure we are evaluating the efficacy of current monitoring networks within state waters, as many funding decisions are made at the state level. To ease computation, we rounded all data to the nearest 0.02 degrees latitude and longitude, pH to the nearest 0.005, and DO to the nearest 0.05% saturation, resulting in over 1.44 million DO data and 1.79 million pH data for exposure calculations. By using previously published dataset and materials, we were not required to obtain permits for this research.

For each species, we set an initial boundary for the latitudinal range. For planktonic larval distributions, we then bounded by depth, distance from shore, and months present, which often differed between northern and southern California populations. For other early life stages (i.e., embryo, benthic megalopae) we bound the analyses by latitude, depth, and months present. For juvenile and adult life stages, which are present year-round, we bound analyses by latitude and depth. For adults, we chose to use the common depth range to ensure our calculations reflect exposure for most organisms, careful to not over-emphasize exposure for individuals found at maximum recorded depths, which may be more tolerant to extreme conditions. With benthic life stages, we used bathymetric data (2022: ETOPO 2022 15 Arc-Second Global Relief Model) to apply an additional boundary that limited oceanographic data to within 20 m of the seafloor. We note in the millions of oceanographic data, few come from near the seafloor below 25 m depth. Conditions at 25 m may be generalizable to the mixed layer [typically 15–75 m; e.g., 75,76], especially when the water column is well-mixed. However, the lack of data at depth limits the accuracy of exposure calculations for deeper-dwelling organisms (i.e., pink shrimp; most adult life stages). As such, for pink shrimp juvenile and adults, we used the above outlined calculation to include near-benthos data (at 80–199 m depth), and also pulled offshore data (from 200–230 m depth). We consider this a reasonable approach for calculating exposure, as pH and DO become more uniform in the nearshore to offshore direction at depths > 200 m [e.g., 18,70].

Grouping oceanographic data by latitude, longitude, year, and day, we generated daily means. Using daily means, we calculated the percentage of weeks that fell below DO thresholds ( < 33%), and months that fell below pH thresholds ( < 7.6). We use weekly percent exposure for DO to reflect the potential rapid response of organisms to hypoxic conditions, and monthly percent exposure for pH to reflect longer response time to more acidic conditions. This distinction also mirrors differences in the design of most of our cited hypoxia studies, which typically examine LC_50_ and SLC_50_ on the order of days to weeks [41,77–83], and acidification studies, which typically examine sublethal effects on the orders of weeks to months [84–89]. However, we acknowledge that larval studies tend to be on the order of days (irrespective of stressor); and there are more experiments examining the effects of low pH on larvae compared to adult life stages, while the opposite is true for low DO.

We then converted percent exposure to an exposure value between 1–3 [28]. We consider any percent exposure greater than 75% high (3), and linearly converted values between 0% and 75% to values between 1–3 [28,30]. We also calculated % exposure at DO = 24, 33, and 39% sat and pH = 7.55, 7.6, and 7.65 to test threshold sensitivity for all organisms and life stages.

### 2.4. Vulnerability

To calculate life stage vulnerability (*V*_*i*_) we multiplied the exposure score (*Ei*, 1–3) by the consequence score (*C*_*i*_, 1–3) to generate a vulnerability score on a scale of 1–9, similar to methods by Berger et al. [30], Halpern [62], and Hodgson et al. [28],


$$V_{i}=(E_{i})x(C_{i})$$ (1)

We also calculated the vulnerability range, defined as the difference between the lowest and highest life-stage vulnerability Hodgson et al. [30]:


Rv=Vmax–Vmin (2)

### 2.5. Uncertainty

To calculate life stage uncertainty in vulnerability (*Uv*_*i*_) we multiplied the uncertainty (*Ue*_*i*_, 1–3) by the uncertainty in consequence (*Uc*_*i*_, 1–3) to generate an uncertainty in vulnerability score on a scale of 1–9, similar to methods by Berger et al. [28], Hodgson et al. [30], and Halpern [62]:


$$Uv_{i}=(Ue_{i})x(Uc_{i})$$ (3)

Without sufficient information to rigorously quantify uncertainty, we followed methods by Hodgson et al. [30 to produce semi-quantitative rankings for uncertainty in exposure (*Ue*_*i*_) and uncertainty in consequence (*Uc*_*i*_) assigning scores on a scale of 1 to 3 [Tables 2 and 3 in S1 File; 28]. Uncertainty estimates follow approaches set by the IPCC, scaling confidence to qualify the conclusions made [Table 1; 90]. Uncertainty in consequence (*Uc*_*i*_) reflects the confidence that an organism’s life stage would respond in the same way as found in laboratory studies. Uncertainty in consequence (*Uc*_*i*_) considers the number of studies, agreement among study responses, and whether the laboratory studies were conducted on the species of interest or instead on a closely, or distantly related species. Uncertainty in exposure (*Ue*_*i*_) incorporates the level of confidence in species and life stage distribution including months present, depth, distance from shore, and latitudinal range, and the spatio-temporal coverage of oceanographic data (Table 4 in S1 File).

**Table 1:** Uncertainty in exposure (*Ue*_*i*_) and consequence (*Uc*_*i*_) scores (left) and the criteria for low (1), medium (2), and high (3) uncertainty (Definition; right).

*Uei*	Definition
1	Low uncertainty: Biogeographic and depth distribution, settlement seasonality and depth distribution, larval seasonality, and distribution (distance traveled from shore, depth) are all well constrained. Conclusions are based on numerous scientific papers or state reports and supported by expert consensus. Moderate-to-High spatial and temporal coverage of oceanographic data.
2	Moderate uncertainty: Biogeographic distribution is based on minimal observations with questionable accuracy of location or local conditions; the full extent of distribution may not be precisely measured or is changing. A few scientific papers or reports loosely constrain larval and settlement seasonality and depth. Low-to-Moderate spatial and temporal oceanographic data coverage.
3	High uncertainty: Biogeographic distribution is not well-known, and is constrained by few, spatially limited papers or reports (1–2). Larval distribution extrapolated from taxa-level studies. Larval seasonality and juvenile settlement rely on papers and reports from outside the region of interest (California). Exposure may be a poor estimate due to behavioral response avoiding extreme conditions. Low or no spatial and temporal oceanographic data coverage.
*Uci*	
1	Low uncertainty: High confidence score based on the number (degree) and agreement among studies. Consequences are derived from experiments on the organism and life stage of interest.
2	Moderate uncertainty: Medium confidence score reflects either several studies (moderate degree of evidence) on studies of the same genus or an individual study on the species and life stage of interest (low evidence, high relevance). Consequence scores that pull from many studies with low agreement will also be given a moderate score.
3	High uncertainty: Low confidence score based on the low degree of evidence and low relevance. Consequences are drawn from a distantly related species, though from the life stage of interest.

### 2.6. Oceanographic data gaps

As previously mentioned, uncertainty in exposure (*Ue*_*i*_) can be heightened by limited literature on biogeographic distribution or by limited oceanographic data. To identify gaps that strongly influence *Ue*_*i*_ *(*for pH and DO), we investigated: 1) the proportion of data coming from above and below the mixed layer, 2) the distribution of data across latitude (0.5 degree bins), 3) seasons, 4) years, and 5) the total amount of data. For planktonic life stages, we also considered distribution of data in the offshore direction. We assigned each coverage type (depth, latitude, season, years, total amount of data) a score from 1 - 3. A score of 1 indicates no gaps, a score of 2 indicates moderate gaps, and a score of 3 indicates multiple, significant gaps. Final *Ue*_*i*_ for each life stage is an average of scores across the 5–6 categories.

## 3. Results

Disparate sensitivity to hypoxic and low pH conditions, differing distributions throughout species life history, and complex regional oceanography create variation in exposure and vulnerability scores across species and life stages. Consequence scores are higher and more variable than exposure scores. Uncertainty in consequence (*Uc*_*i*_) is heightened by absence of laboratory experiments on closely related species. The highest life stage exposures (>10%) are sensitive to changes in selected DO and pH thresholds, depth range (common vs maximum), and regional and seasonal oceanography. Large latitudinal data gaps, sporadic data collection, and a recent overall decline in monitoring limit certainty in exposure (*Ue*_*i*_) for most species. Exposure to either stressor is low for most life stages, and thus, consequence scores exert more influence over the final vulnerability scores. Below we describe consequence and exposure scores; exposure sensitivity to selected biological thresholds, temporal averaging, and seasonality; vulnerability, and oceanographic data gaps.

### 3.1. Uncertainty in consequence scores

Uncertainty in consequence scores differ significantly among species and life stages due to the number of studies conducted on the species and life stage of interest, and the degree of consistency in organisms responses to stressful conditions (Tables 2 and 3 in S1 File). Numerous studies investigate the effect of acidification on Dungeness crab, and are in high agreement about sublethal impacts, resulting in low *Uc*_*i*_ for both DO and pH consequences (Tables 2 and 3 in S1 File). By contrast, the numerous studies on purple urchin, particularly larval response to low pH conditions, disagree on their degree of sensitivity to low pH conditions, resulting in a higher *Uc*_*i*_. There are multiple but fewer studies on red urchin, whose *Uc*_*i*_ is heightened by evidence of adaptive maternal responses alongside larval responses to low pH conditions [44], and because there are only multistressor studies examining effects of pH and DO on juveniles [temperature, DO, pH; 73,91], making it unclear whether individual stressors would result in sublethal responses.

Despite using fewer studies to develop pH thresholds, there are more studies examining the impacts of pH on CCS species relative to studies examining the impact of low DO, and as such, *Uc*_*i*_ for DO generally exceeds *Uc*_*i*_ for pH. Adult Dungeness crab is the only life stage with numerous studies examining its response to hypoxic conditions. By contrast, we were unable to find any studies examining the effects of low pH, low DO conditions on any spiny lobster or lobster species, inhibiting us from assigning consequences, and thus vulnerability. There are no known studies examining the effects of acidification on warty sea cucumber, or any sea cucumber that inhabits the CCS, resulting in high *Uc*_*i*_ values across all life stages. In several instances we assumed juvenile responses to low DO, low pH would be the same as adults, as no studies were conducted on juveniles, resulting in high uncertainty scores.

### 3.2. Exposure scores and sensitivity to selected thresholds and depths

**Fig 3:**
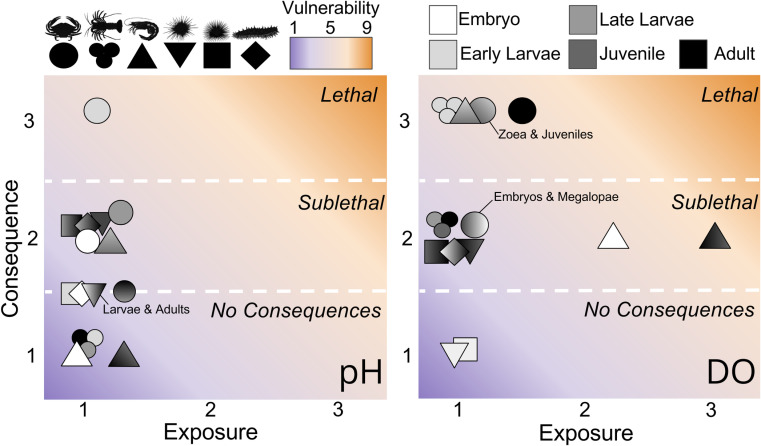
Consequence, exposure, and vulnerability scores for each life stage to low pH (<7.6) and low DO (<33% sat). Vulnerability is the product of the consequence and exposure scores and is colored from blue (1) to orange (9). Organisms are represented as symbols, including Dungeness crab (circle), California spiny lobster (trinity), pink shrimp (triangle), red urchin (inverted triangle), purple urchin (square), sea cucumber (diamond) are plotted relative to their exposure and consequence for dissolved oxygen (left) and pH (right). Dashed lines and text denote when we expect no consequences, sublethal, or lethal consequences from calculated exposure. Shades denote life stage, including embryo (white), zoea and larvae (light grey), megalopae and late larvae (medium grey), juveniles (dark grey), and adults (black). Where multiple life stages have the same scores there is a gradient or mixture of colors. Where no text labels are present these are subsequent life stages (i.e., embryo & larvae, juvenile & adult). In instances where they are not subsequent life stages, text labels denote which labels are represented by that symbol.

Exposure to low DO is less common than exposure to low pH. All organisms have < 10% exposure to pH < 7.6 resulting in exposure scores that range from 1–1.2 (Tables 5 and 7 in S1 File; Fig 3). Juvenile and adult pink shrimp have the highest exposure (9.2%) followed by adult Dungeness crab (8.4%). All organisms except pink shrimp and adult Dungeness crab have < 4% exposure to DO < 33% sat, resulting in exposure scores of 1–1.1. Yet, exposures for adult Dungeness crab (19.6%, 1.5) pink shrimp embryos (39.6%, 2.1), and juveniles/adults (73.4%, 3.0) are so high that they make the average exposure to DO < 33% (9.2%) exceed the average exposure to pH < 7.6 (3.5%), despite the median exposures being 0.1% and 3.5% respectively.

Only the highest exposures are sensitive to changing thresholds. As pH thresholds move from 7.55 to 7.65, Dungeness crab adults and pink shrimp juveniles/adult exposure scores move from 2.5% to 15.1%, and from 4.3% to 25.7%5, respectively (SI, Table 6 in S1 File; S1 Fig). Their DO exposures are even higher, and thus more sensitive to changing thresholds. Moving the DO threshold from 24% to 39% sat shifts adult Dungeness crab exposure from 6.5% to 34.8%, pink shrimp embryo exposure from 14.2% to 54.9%, and juvenile/adult pink shrimp exposure from 25.1% to 90.4% (Table 8 in S1 File; S1 Fig ).

Adult exposures also have varying sensitivities to the depth range selected for exposure calculations; proportional to the difference between their common and full-depth ranges (Table 9 in S1 File). The most dramatic difference is for adult red urchins’ exposure to low DO, which shifts from 1.1% within the common range (0–50 m) to 49.5% within the full depth range (0–284 m; Table 8 in S1 File). Similarly, but less dramatically, Dungeness crab increase in exposure to low DO from 19.6% in their common range (30–90 m) to 34.4% in their full depth range (0–220 m). This percent change is on par with pink shrimp, despite them having the largest difference between common (80–230 m) and full (0–1400 m) depth ranges. Within the common depth range, pink shrimp are exposed to low pH 9.2% of the time, and low DO 73.4% of the time, whereas within the full depth range they experience low pH and low DO 26.3% and 92.5% of the time, respectively (Table 9 in S1 File). There is no difference between warty sea cucumber and California spiny lobster common and full depth ranges, and thus, no difference between exposure scores (Table 9 in S1 File).

### 3.3. Exposure across seasons and biogeography

Looking exclusively at adult life stages, which are present year-round, nearly all exposures peak in the summer (S2 Fig). Seasonal differences are dramatic for some species, including Dungeness crab, which experience 29.5% exposure to low DO and 12.9% exposure to low pH in the summer, and no exposure to either stressor in the fall and winter. Similarly, pink shrimp adults experience 81.8% exposure to DO in the summer months compared to 68.1–70.5% in fall and winter; and 12.9% exposure to low pH in summer compared to 0% in fall and winter. For organisms with low exposures there is little difference between seasons. However, these interpretations are also limited by data availability. For example, there is no data collected at appropriate depths for warty sea cucumber that travel offshore in the summer months, inhibiting a true comparison across all seasons.

Comparing exposure between species in the northern (>37.7°N) and southern (<37.7°N) halves of coastal California (red urchin, purple urchin, pink shrimp) reveals that shallower-dwelling species and life stages experience more exposure to low pH in Southern California, and more exposure to low DO in Northern California, while deeper dwelling organisms and life stages experience more exposure to low DO in Southern California (Table 10 in S1 File). Biogeographic differences are subtle for red and purple urchin at all life stages, who do not exceed 50 m depth, with < 4% more exposure to pH in the south, and < 4% more exposure to low DO in the north. Biogeographic differences in dissolved oxygen exposure are more dramatic for the deeper dwelling pink shrimp embryos, juveniles and adults, who inhabit up to 230 m, with 17.9–37.5% more exposure to low DO in Southern California, relative to Northern California. Contrasting their exposure to low DO, there is little difference in their pH exposure between the northern and southern regions.

### 3.4. Data availability and uncertainty in exposure

**Fig 4:**
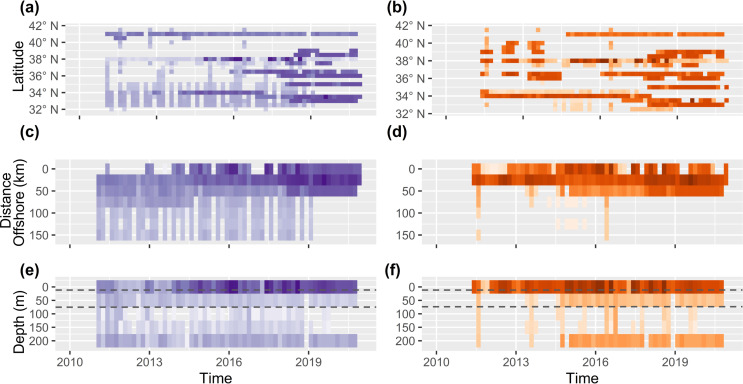
Frequency of oceanographic monitoring along the California coast through time and space. The number of oxygen (left) and pH observations (right) at < 250 m and < 100 km from shore along in the last decade. Data coverage is viewed across latitude (a, b) and distance offshore (c, d), and the number of near-bottom (<25 m from seafloor) oxygen (e) and pH (f) observations across depth. Dashed line denotes the minimum and maximum mixed layer depths.

Oceanographic data gaps heighten our uncertainty in exposure, and thus vulnerability. The spatial coverage of oceanographic observations in California has declined between 2016 and 2021 [61]. Most oceanographic data collection occurs at 3 latitudes: 34.5°N, 38°N, and 41°N. Though the MOCHA database is not exhaustive, the apparent absence of DO data collection from 34.7 - 36°N and 38.5 - 41°N are sources of uncertainty in most benthic life stages (Fig 4a). Likewise, few pH data are collected from 38.5 - 41°N outside of summer months, in offshore, or near the benthos, heightening uncertainty in exposure for most life stages of pink shrimp and Dungeness crab. In southern California there is almost no pH or oxygen data collection from 3–12 km offshore (Fig 4c and 4d), and no pH observations from > 10 km offshore, heightening uncertainty in exposure scores for most larval life stages. The deficiency of near-bottom data collection at depths of> 15 m heightens uncertainty in exposure for most benthic life stages (Fig 4d and 4e). Beyond these general gaps, some species and life stages data have minimal data for exposure calculations. For example, the vast majority of data for Dungeness crab late zoea and pelagic megalopae exposure calculations come from 41°N (Tables 11 and 12 in S1 File). Likewise pink shrimp embryos only have data from 41°N in the northern half of their distribution, and only offshore, pelagic data at> 200 m depth collected in May 2016 in the southern half of their distribution (Tables 11 and 12 in S1 File). Most data for all California spiny lobster life stages comes from 34.5°N. These data gaps all lead to higher *Ue*_*i*_ (Tables 11 and 12 in S1 File).

### 3.5. Vulnerability and uncertainty

While vulnerability scores could theoretically reach 9, our maximum V_i_ scores are just above 6, reflecting either low to moderate exposures with high potential consequences or high exposures with low potential consequences. For example, Dungeness crab adults experience low DO relatively frequently (25%, *E*_*i*_ = 1.7) and are predicted to have a lethal response to these conditions (*C*_*i*_ = 3), resulting in a *Vi* of 5.1. Whereas pink shrimp embryos, juveniles, and adults frequently experience low DO (43.6%, 82.4%. *E*_*i*_ = 2.2 and 3) but are only predicted to experience sublethal consequences, such as decreased yolk size and delayed hatching for embryos and slower growth and feeding rate in adults (*C*_*i*_ = 2), resulting in *V*_*i*_ of 4.4 and 6. Given pink shrimp and Dungeness crab’s high vulnerability to low DO, they also have the highest range in vulnerability scores, with *Rv =* 3.4 and *Rv =* 3.1, respectively. Red and purple urchin larvae, and all life stages of California spiny lobster have the lowest vulnerability scores, with *V*_*i*_ = 1 for DO.

Most organisms have relatively low exposure to pH, low oxygen conditions, so consequence scores have more influence over most organisms’ vulnerability scores (Tables 13 and 14 in S1 File ). In several cases, exposure scores and anticipated consequences are both minimal, leading to *V*_*i*_ = 1, the lowest possible vulnerability score. This is the case for California spiny lobster megalopae, juveniles, and adults vulnerability to low pH; pink shrimp embryos vulnerability to low pH; and red and purple urchin vulnerability to DO at all life stages. For Dungeness crab juveniles and adults, California spiny lobster zoea, and pink shrimp juveniles and adults, vulnerability scores are slightly higher since minimal consequences (*C*_*i*_ = 1) are paired with low, but present, exposures, resulting in *V*_*i*_ up to 1.2. The same is true for pink shrimp embryo vulnerability to low DO. Pink shrimp embryos, juveniles, and adults are the only life stages with exposure scores that substantially exceed their consequence scores (*E*_*i*_ = 3, *C*_*i*_ = 2).

Comparing vulnerability to acidification and low oxygen across all species and life stages, average DO *V*_*i*_ is generally higher because of higher consequence scores. Specifically, average *E*_*i*_ for low DO and pH are 1.3 and 1.1 respectively, while average *C*_*i*_ are 2.2 and 1.6. At first glance these scores suggest that while most life stages infrequently experience either stressor, they are more likely to suffer physiological consequences in response to low oxygen conditions. These patterns hold true for most pink shrimp and California spiny lobster life stages and for juvenile and adult Dungeness crab. However, for both sea urchins, larvae have demonstrated tolerance to low oxygen and possibly low pH conditions, and all life stages occupy depths that are typically oxygen saturated on the coast. Thus, generally speaking, decapods are more vulnerable to deoxygenation than acidification, while urchins are more vulnerable to acidification than deoxygenation. By contrast, Dungeness crab embryo, and megalopae are equally vulnerable to acidification and deoxygenation, and larvae are slightly more vulnerable to acidification (*V*_*i*_ = 3.3 vs. *V*_*i*_ = 3.0) while warty sea cucumber larvae are slightly more vulnerable to deoxygenation than acidification (*V*_*i*_ = 2.0 vs *V*_*i*_ = 1.7).

**Fig 5:**
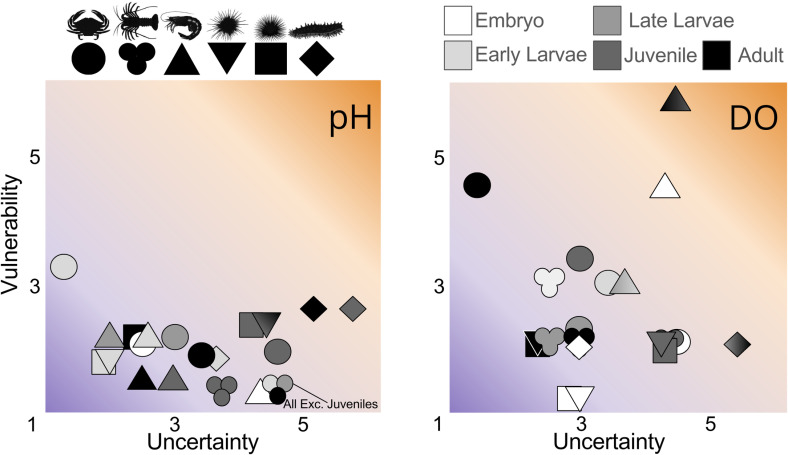
Vulnerability and uncertainty in vulnerability scores for low pH (<7.6) and low dissolved oxygen (33% sat). Scale is plotted with maxima of six to represent the range values, though vulnerability and uncertainty could hypothetically reach maxima of nine. Organisms are represented as symbols, including Dungeness crab (circle), California spiny lobster (trinity), pink shrimp (triangle), red urchin (inverted triangle), purple urchin (square), sea cucumber (diamond), are plotted relative to their exposure and consequence for dissolved oxygen (left) and ph (right). Shades denote life stage, including embryos (white), zoea and larvae (light grey), megalopae and late larvae (medium grey), juveniles (dark grey), and adults (black). Where multiple life stages have the same scores there is a gradient or mixture of colors. Where no text labels are present these are subsequent life stages (i.e., embryo & larvae, juvenile & adult). In instances where they are not *subsequent* life stages, text labels denote which labels are represented by that symbol.

Uncertainty in vulnerability is high and variable among species and life stages (Fig 5; Tables 15 and 16 in S1 File ). Average *Uc*_*i*_ is higher than *Ue*_*i*_ (*Uc*_*i*_ = 2.4–2.6*, Ue*_*i*_ = 1.4), suggesting that the lack of relevant laboratory studies impacts confidence in vulnerability scores more than a lack of information on species distribution or coverage of oceanographic data. Juveniles and embryos generally have the highest *Uc*_*i*_, *Ue*_*i*_*,* and thus *Uv*_*i*_ scores, stemming from their limited use in experiments, and limited knowledge on their depth distribution (Table 4 in S1 File ), partially explained by their use of cryptic habitats [92–94]. Shifting to a species perspective, warty sea cucumber have the highest *Uv*_*i*_ for both low DO and pH (5.25, 6, respectively) driven by a potent combination of no direct studies on the consequences acidification or deoxygenation on this species, a mismatch between their distributions at several life stages and the density of oceanographic monitoring, and several unknowns about their life history and depth distribution (Table 4 in S1 File ).

## 4. Discussion

Benthic invertebrates’ vulnerability to ocean acidification and deoxygenation changes throughout life history, influenced by life stage sensitivities and varying exposure across dynamic habitat use. Our reported exposures are significantly lower than those previously reported for organisms in the northern CCS [30] and those predicted for the future central CCS [28]. With limited exposure rates across all organisms and life stages, physiological sensitivities and resultant consequences exert a stronger influence over species vulnerability. Most life stages experience low pH conditions more frequently than low oxygen conditions. Exposure to either stressor is generally low, however, except for life stages occupying the greatest depths. The number of oceanographic observations available generally peaks in summer, when organisms generally experience the highest exposure to both stressors. This seasonal bias combines with significant spatial and benthic gaps in monitoring efforts, with important implications for resource management and constraints on vulnerability.

### 4.1. Exposure risk to low pH, low oxygen conditions

We find that deeper-dwelling organisms in the central CCS are exposed to stressful conditions more frequently than those in shallow waters, consistent with known declines in pH and DO with depth [18,69,95]. Below the mixed layer, which is shallowest in summer (~15 m) and deepest in winter [~75 m; 75,76]; atmospheric mixing cannot as readily replenish oxygen, nor ventilate waters enriched with dissolved inorganic carbon from upwelled waters or respiration. Organisms that do not occupy surface waters ( <30 m), such as adult Dungeness crab and pink shrimp juveniles and adults frequently experience low DO, low pH conditions within their common depth range. These life stages also frequently experience pH and oxygen conditions just above threshold values (Tables 5 and 7 in S1 File), suggesting their exposures will substantially increase with near-future declines of oxygen and pH [i.e., 28,30,56]. However, at least for pink shrimp, populations at the maximum extents of their depth ranges already frequently experience conditions that surpass biological thresholds (Table 9 in S1 File), which could confer resilience as ocean acidification and deoxygenation progress.

Seasonal variation in exposure is most dramatic for organisms that do not occupy surface waters, and particularly for at depths where conditions hover around threshold values. Exposure to hypoxic and acidic conditions peaks in summer for all life stages (S2 Fig). However, for shallow dwelling organisms and life stages (<80 m), particularly those occupying surface waters, seasonal fluctuations are minimal. Seasonality is highest for adult Dungeness crab, occupying depths of 30–90 m, where summer exposures are 13% higher for pH and up to 29.5% higher for DO. Adult pink shrimp, who occupy even greater depths (80–230 m) where DO conditions are consistently low [i.e., 70], see a muted seasonal fluctuations in DO, with ~ 10% higher exposure to low DO conditions in the summer, and a similar degree of seasonal fluctuation in pH as seen for adult Dungeness crab. Life stage-speific exposure shows that for the shallowest and deepest-dwelling organisms somewhat agrees with previous assumptions that year-round upwelling off the California coast would result in comparable seasonal and year-round exposures [30,96], at least in terms of exposure scores, which hit a maximum of *E*_*i*_ = 3 at 75% exposure. However, adult Dungeness crab seasonal exposures to low DO conditions significantly differ from one another, though they do not differ as strongly from year-round exposure as calculated here, due to data collection bias towards spring and summer months. Though seasonal and annual scores were relatively similar for most organisms in this study, exposure calculations that equally weight data from throughout the year may underestimate the physiological consequences of chronic seasonal exposure, particularly if there is limited recovery time between subsequent events [e.g., 42,97–101]. Future vulnerability assessments for benthic invertebrates would benefit from examining seasonality, irrespective of perceptions regarding the degree of seasonality in the region.

### 4.2. Oceanographic data gaps and uncertainty in exposure

Though California is home to one of the most comprehensive oceanographic monitoring networks, latitudinal data gaps pose significant challenges in estimating species vulnerability to ocean acidification and deoxygenation. Latitudinal data gaps are the most common source of uncertainty in DO exposure calculations, and second greatest source of uncertainty in pH exposure calculations. Aside from 2016 when monitoring coverage peaked, there are consistently hundreds of kilometers of unsampled coastline (Fig 4a). The largest and most common latitudinal data gap in our exposure calculations is from 38.5 - 41°N, and to a lesser extent 34.5–36°N, particularly for benthic life stages (Tables 11 and 12 in S1 File). Sporadic spatial coverage makes it challenging to accurately estimate species range of exposure to low pH, low DO conditions, which is highly variable across small spatial scales [e.g., 21,61,71,101–104].

Despite comprehensive ship-based and sensor-based monitoring efforts there is a persistent deficiency of publicly available near benthos data collection, which can lead to severe underestimates in exposure and high uncertainty. In California, only two sensors gather publicly available pH and oxygen data at greater than 15 m [61], and while oceanographic cruises collect data down to thousands of meters depth, they rarely sample near-bottom conditions, given the high risk of damaging or losing equipment. As previously discussed, juvenile and adult benthic invertebrates face the most exposure to acidic and hypoxic conditions below the mixed layer. Data collected nearshore, within the mixed layer at a given location are likely representative for conditions throughout the entire mixed layer at that location. However, those conditions cannot be extrapolated to depths greater than the mixed layer. Similarly, data collected offshore, far from the benthos, at depths < 200 m cannot be extrapolated to similar depths in nearshore, benthic environments [e.g., 18,70]. For organisms whose depth ranges exceed 200 m (i.e., pink shrimp embryos, juveniles, and adults), we can reasonably incorporate offshore, pelagic data at> 200 m, which become the only data from such depths in their exposure calculations. For other benthic life stages, we find that a near absence of benthic data at depths > 15–30 m is the most common cause of uncertainty in exposure calculations. By failing to monitor the benthos at depth, we have the least data collection in locations where benthic organisms are the most likely to experience high exposure to acidic, and hypoxic conditions. Though we still calculate vulnerability scores for the life stages affected by benthic data gaps, the scores are uncertain, potentially limiting their use by resource managers.

Oceanographic sampling in California, especially ship-based sampling, is biased towards spring and summer (S3 Fig), posing unique challenges for evaluating different life stages’ exposure to acidic, hypoxic conditions. Ship-based sampling is one of the few options for capturing conditions experienced by deeper dwelling life stages that do not occupy surface waters (0–25 m), and early life stages that travel far offshore. Though ship-based sampling efforts intentionally set out in spring and early summer to capture maximum exposure to stressful conditions, a bias towards spring and summer data could result in overestimates of annual exposure and vulnerability for life stages at depth, and offshore (S2 and S3 Figs). Temporal sampling biases pose an ever greater challenge for early life stages that are not present during peak sampling. For example, Dungeness crab embryos, which are present from November to February, have few data for their calculations despite occupying nearshore waters at 0–20 m depth. Pink shrimp embryos, which are present from October to May and do not occupy surface waters, have few data for their calculations, with all northern California pH data coming from the Trinidad Head Line, and all southern California data coming from the 2016 West Coast Ocean Acidification cruise [61].

### 4.3. Considerations for management

Species and life stages investigated herein significantly differ in their exposure to ocean acidification and deoxygenation, existing monitoring coverage across their distribution, overlap of fishing effort with the season of maximum stress, and the degree of environmental data considered in management decisions. For most species and life stages investigated herein (i.e., spiny lobster, sea cucumber, red, and purple urchin), current exposure and vulnerability to ocean acidification and deoxygenation are likely too minimal to warrant immediate management action, though the potential impacts of ocean acidification are making their way into some management plans (i.e., California Spiny Lobster). For species with life stages that experience high, and strongly seasonal exposure to ocean acidification and deoxygenation, or severely lack data for such assessments, precautionary principles and proactive management strategies may be necessary to avoid over-extracting from vulnerable populations [e.g., 105,106]. Unfortunately, at present, ocean acidification and deoxygenation are not explicitly considered in harvest control rules, or essential fisheries information (EFI) data collection, despite well-documented impacts on bottleneck life history stages and anticipated impacts on feeding, reproduction, growth, and thus age at maturity, and survival; all of which fall within EFI categories and priorities set by the Master Plan. Here we provide two examples, pink shrimp and Dungeness crab, of how management might incorporate oceanographic data pertaining to ocean acidification and deoxygenation in adaptive management strategies.

California’s pink shrimp fishery management plan considers sea level height (SLH) in Crescent City from April of the previous year to January of the current year, and average June landings from the previous year when setting the annual harvest control rule (HCR). Low SLH correlates with upwelling and strong recruitment [107]. Yet, in spring and summer, when upwelling events are frequent, pink shrimp experience nearly constant exposure to low pH, low DO conditions (S2 Fig ), especially at depths of 200 m or more. If physiologically stressful conditions persist, it may compress their habitat, as seen with other species [e.g., 37,108–110]. Migration into more commonly fished depths of 90–180 m could inflate the June catch, leading to an over-exploitative HCR. Though low exposure in fall and winter may provide adequate recovery time, with known hindrances to hatching success and survival at all life stages; and a young age of maturity (1–3 years-old), it is reasonable to suggest that large harvests of an already stressed population could impact landings over time. To investigate relationships between seawater chemistry and June landings, pink shrimp managers could examine spring pH and DO data from monthly cruises along the Trinidad Head Line [seventy miles south of Crescent City, the site of sea level height calculations; 111], and collaborate with researchers to investigate whether pink shrimp avoid low pH, low DO conditions. If DO and pH conditions are low enough to evoke sublethal or lethal effects, and there is evidence that pink shrimp would migrate to avoid stressful conditions, the fishery could choose a slightly more conservative HCR given the potential stress on the population, and to reflect the possible inflation of catch size due to habitat compression.

Contrasting the pink shrimp example, Dungeness crabs are mostly fished in fall and winter when available data indicates exposure is low, with the spring portion of the season increasingly truncated by concerns around whale entanglement [e.g., 112–114]. Sparse data availability from November through March greatly limits understanding of whether and how low pH and low DO impact the fishery (S3 Fig). In Oregon, where hypoxic events (<2.0 mg L^−1^) have clearly and dramatically impacted the Dungeness crab fishery, collaborative partnerships have expanded monitoring efforts by installing oxygen sensors on crab pots that relay concentrations to fishermen and researchers in real-time, such that they move their crab pots if oxygen levels drop below critical thresholds [115]. Similar efforts could improve oxygen monitoring coverage in California, though there are no equivalent sensors for carbonate chemistry at this time. Though such data would improve our understanding of the fishery’s vulnerability to hypoxic events, and adaptation by individual fishermen, it is unclear how the current management framework would incorporate environmental data, as historically, the fishery has only closed in response to human health risks (domoic acid) and conservation concerns (whale entanglement). Managers may want to consider ways to incorporate more environmental data, particularly considering that other dominant stressors (i.e., whale migration, domoic acid) are often related to the same processes that create low pH, low oxygen conditions [i.e., upwelling, stratification; 116–119].

### 4.4. Comparison to other studies

Relative to Berger et al.’s (2021) investigation of Dungeness crab vulnerability in the north California Current System [N-CCS; 30], we found lower and differing exposure to acidic, hypoxic conditions in our study of the central California Current System (C-CCS). In part, this is because we use a lower pH threshold of 7.6 compared to their thresholds of 7.65, which would result in lower exposures even if conditions were identical. Likewise, our DO threshold of 33% sat represents a concentration range of 2.6 mg L^−1^ to 4.0 mg L^−1^ across the vast majority temperature and salinity conditions experienced in Oregon and Washington [T = 4–21°C, S = 20–35; 61], all of which are higher than the Berger et al. DO threshold of 2.0 mg L^−1^. However, aside from our differing thresholds, we generally expect higher exposure to both stressors for embryos, juveniles, and adults in the N-CCS than the C-CCS as it has lower benthic pH, and more frequent hypoxic [e.g., 95,120–123].

Even using a higher pH threshold of 7.65 (Table 6 in S1 File ), our reported exposures for Dungeness crab and pink shrimp remain far below predictions for the year 2050 for the C-CCS [also used 7.65 threshold, 28]. However, our study does not include Oregon where benthos experience acidic conditions more frequently than that of California [e.g., 95,122–123]; and we calculate exposure using data from across organisms’ depth distributions rather than exclusively using conditions at maximum depth. The only life stage with vulnerability that approaches values presented by Hodgson et al. is Dungeness crab megalopae [1.2 at a threshold of 7.65 (Table 6 in S1 File ), 1.43 in Hodgson et al., 2016; 28]. A potential concern is that sooner than predicted sublethal and lethal effects in earlier life stages could bottleneck population recruitment and growth, which could be underestimated in vulnerability assessments that assign low weights to early life stages with high mortality rates [79,124–126].

Looking towards far future (2100) conditions across the CCS, Sunday et al. predict the manifestation of impacts that may already be occurring at current conditions [56]. Their scaled sensitivities predict a decline in red urchin growth and survival in response to acidification; and declines in Dungeness crab growth, consumption, and survival in response to end-of-century acidification and deoxygenation across the California current. Their conclusion that Dungeness crab and red urchin will experience sublethal and lethal impacts generally agrees with the findings of our study.

### 4.5. Benefits and drawbacks of our approach

In using oceanographic data and attempting to incorporate conditions experienced by organisms across their common depth range, we may underestimate exposures relative to other studies. For life stages that experience many of the previously outlined data gaps, our calculations may not accurately represent exposure across their depth, and biogeographic range. For organisms that commonly occupy surface waters we chose to include data from throughout the water column, as it reflects the range of conditions experienced across depth distributions. However the shallow bias of observational data creates lower exposures than exposure calculated at maximum depth [e.g., 28,31–32] or along the benthos [e.g., 30] as is used by most vulnerability assessments. Similarly our use of common depth ranges minimizes estimated exposure to hypoxic and acidic conditions. For multiple study species their maximum depth range exceeds their common depth range, like pink shrimp whose full adult range of 0–1400 m far exceeds their common range of 80–230 m, and Dungeness crab whose full range of 0–220 m exceeds the common range of 30–90 m. Conditions across the maximum depth range may be comparable to, or far exceed the common range (Table 9 in S1 File ). We note that there are other depth ranges to consider as well, such as the range of fishing depths which strongly depend on the equipment and approach used by the fishery. In choosing the common range for our calculations, our reported exposures and vulnerabilities are relevant for the majority of the population. We encourage future vulnerability assessments to consider exploring alternative depth ranges, and to consider reporting integrated scores from across their depth range alongside maximum depth exposure and vulnerability, to allow for more robust comparisons.

In using oceanographic data to calculate weekly percent exposure to low DO conditions, and monthly percent exposures to low pH conditions, we are able to examine stressors on physiologically relevant timescales at the expense of examining the spatio-temporal overlap of low pH, low oxygen conditions. The multistressor framework assumes that stressors are experienced at the same time [127]. Studies that derive ocean conditions from the Regional Ocean Model System simulations, or other oceanographic model simulations, are able to examine the co-occurrence of stressors because they have pH, DO, and temperature data of equal spatial and temporal resolution [e.g., 30,56]. Given the patchwork nature of oceanographic monitoring [61], our DO and pH data have very different spatial and temporal resolution, and most measurements are not taken concurrently. To assess multiple stressors using oceanographic data, researchers must either limit analyses to locations where there is simultaneous monitoring of DO and pH [e.g., 72], or use an exposure metric that eliminates the element of time, such as intensity, defined as the negative magnitude of the departure from the threshold [e.g., 31–32]. However, limiting our analysis to co-measured data would greatly limit data availability to assess species and life stage exposure to stressors across the state of California over a period of 12 years, resulting in much higher uncertainties, and likely the exclusion of life stages who may have no contemporaneous pH and DO monitoring within their distributions. Even with improved oceanographic monitoring, there are insufficient studies examining the concurrent impact of low pH and low DO stress [e.g., 73,91,128], to accurately assess their cumulative sensitivity and vulnerability [30].

With respect to consequences, the accuracy of our predicted responses is limited by the number of studies conducted on species and life stages of interest, and closely related species. Ideally, sensitivity studies across a range of plausible low pH or low DO conditions would be available for each species and life stage of interest, providing a continuum of null, sublethal, and lethal responses that could be used to accurately assess species vulnerability [e.g., 28,30,56]. Unfortunately for many organisms, especially those of lesser economic or conservation interest (i.e., warty sea cucumber), there are few or no studies, even on closely related species (Tables 2 and 3 in S1 File ). Likewise, there is a bias towards life stages that are perceived to be more vulnerable to stressors, namely larval life stages for low pH, and adult life stages for low DO. When there are no studies on a species of interest, or no studies on a specific life stage, it is less clear whether individual studies on distantly related species provide more predictive outcomes than using thresholds that reflect sensitivities at a broader taxonomic level [e.g., 31,32]. Even in instances where we use studies on closely related species from different habitats (i.e., shallow vs deep), this mismatch may lead to over-, or underestimates of species sensitivity and vulnerability. These limitations highlight the need for more experimental work that examines the effects of low pH, low oxygen conditions on understudied California Current species.

Current vulnerability assessment methods for calculating uncertainty in consequence scores interpret disagreement among studies as a source of error or uncertainty [44], when in fact it may actually be a sign of genetic variability that would enable adaptation, and thus resilience to changing ocean conditions. For example, some studies show strong, negative impacts on larval purple urchin growth and development [43,46,49], while others show equally strong effects [48,54], or no effects [50–52] and evidence for adaptive genomic and phenotypic responses to acidified conditions, particularly among offspring of populations near strong upwelling centers. Here, we assign this life stage consequence a moderate confidence score, despite the number of well-crafted studies,because of the disagreement among the results. However, as the only life stage with numerous studies examining the response of populations that exist along a mosaic of low pH conditions, it is highly possible, if not likely, that similar studies on other species, life stages, and stressors would yield equally nuanced results, as seen with the comparatively smaller body of literature on juvenile red urchins [74,91]. For warty sea cucumber and California spiny lobster, uncertainty stems not from a plethora of studies, but a lack of them, leaving no opportunity to consider local adaptation (Tables 2 and 3 in S1 File). With the current literature available, it is not possible to incorporate evidence of local adaption and the resilience it confers in a more robust way, within this, and other vulnerability frameworks [129]. Future vulnerability assessments would greatly benefit from more studies that explore local adaptation to low pH and DO conditions, on a wider variety of species and life stages.

## 5. Conclusions and directions for future work

Though there are still significant unknowns in benthic invertebrates’ vulnerability to ocean acidification and deoxygenation, here we observe clear patterns in exposure and vulnerability. Life stage and species distribution are important factors to species exposure. However, with such low overall exposure, physiological sensitivity ultimately drives vulnerability. Our results highlight how laboratory experiments examining the impacts of low DO on multiple life stages, better-constrained species distributions, and targeted and sustained monitoring efforts could improve our understanding of vulnerability for many ecologically and economically valuable benthic invertebrates across the state.

One source of uncertainty not widely discussed in marine ecological vulnerability assessments is a lack of detailed information and formal consensus on species depth distributions (Table 4 in S1 File ). The amount of detailed information on life stage and species distributions strongly depends on their economic value and the type of fishing equipment used in their extraction. For example, Dungeness crab and pink shrimp fisheries that employ traps and nets have accurately constrained the common depth range of these species, compared to warty sea cucumber, whose distributions are quite elusive. Irrespective of using oceanographic data or models, more detailed information on species distributions is needed for accurate vulnerability calculations.

Adequately assessing benthic invertebrates exposure and vulnerability to ocean acidification and deoxygenation will require a concerted and critical funding and sampling effort. We encourage future monitoring efforts to prioritize fishing areas, depths, and open seasons while addressing latitudinal data gaps, and prioritizing benthic sampling at relevant depths.

Alongside advocating for enhanced monitoring and continuous improvement of species vulnerability assessments, we also want to emphasize the need for targeted use of environmental data in fisheries management decisions. Historically, environmental data is only incorporated into harvest control rules when there is a strong, predictable, and positive relationship between recruitment and an environmental factor (i.e., temperature, upwelling); and still, only in species with short pre-recruit survival windows and life history bottlenecks [130]. Though pH and DO conditions may not warrant immediate management action, we highly recommend managers consider how vulnerability assessments and oceanographic monitoring could inform adaptive management strategies (i.e., HCR) before conditions more frequently surpass biological thresholds. Further, we suggest that funding and management agencies collaborate with fishermen to address persistent data gaps, as their direct involvement would enhance transparency in decision-making and facilitate flexibility in fishermens’ response to changing ocean conditions [i.e., 115,131].

## Supporting information

S1 File Information regarding species distributions, life stage consequences, exposures, and vulnerability to low pH, low dissolved conditions, and uncertainty associated with those calculations. (DOCX)

S2 File Treatment conditions from studies examining the effects of low pH, low oxygen conditions on species of interest to this study, or closely related species. (XLSX)

S1 Fig Average percent exposure across all species’ life stages, calculated across a range of pH (left) and dissolved oxygen (DO) thresholds (right). The dotted lines represent low and high pH and dissolved oxygen thresholds (7.55 and 7.65 for pH; 24% and 39% saturation for DO). The dashed line represents the thresholds used in this study (7.6 for pH, 33% saturation for DO). For pH, percent exposure is calculated as the number of monthly averages falling below the threshold. For dissolved oxygen, percent exposure is calculated as the number of weekly averages falling below the DO threshold. Exposures for *P. jordani, C. magister* increase significantly between the low and high thresholds.
(PNG)

S2 Fig The distributions of percent exposure to dissolved oxygen < 33% saturation and pH < 7.6 by season for adult life stages. Winter includes data from January to March, spring includes data from April to June, summer includes data from July to September, and fall contains data from October to December. For P. interruptus, C. Magister, P. jordani, percent exposure is calculated as the number of monthly averages falling below the pH threshold. For M. franciscanus, S. purpurpatus, and A. parvimensis percent exposure is calculated as the number of weekly averages falling below the pH threshold. For all organisms percent exposure to dissolved oxygen is calculated using weekly averages. Exposures for P. jordani, and C. magister increase significantly between the mid and high thresholds.
(PNG)

S3 Fig The distributions of unique dissolved oxygen and pH observations by season adult life stages, with an equal split represented by dashed lines (25%). Winter includes data from January to March, spring includes data from April to June, summer includes data from July to September, and fall contains data from October to December. Spring and Summer pH observations are overrepresented, while Fall and Winter are underrepresented, with the exception of A. parvimensis (California warty sea cucumber), which moves from onshore to offshore in the summer months. Fall is underrepresented in dissolved oxygen observations, with a slight overrepresentation of other seasons.
(PNG)
