# Supplementary material for: Assessing benthic invertebrate vulnerability to ocean acidification and de-oxygenation in California: The importance of effective oceanographic monitoring networks
Source: PLoS One. 2025 Feb 18;20(2):e0317906. doi: 10.1371/journal.pone.0317906 (PMC11835291; doi:10.1371/journal.pone.0317906)
Supplement: S1 File — (DOCX) [file pone.0317906.s001.docx]

Table 1. Biogeographic and depth distributions and life history information used to constrain exposure calculations

| **Species** | **Life stage** | **Assumptions** | | **Citations** |
| --- | --- | --- | --- | --- |
| *Apostichopus*  *(Parastichopus)*  *parvimensis*  Present from Puerto San Bartolome, Baja California, Mexico, through Fort Bragg, California, US, though they are most common south of Monterey, California, US (Chavez et al., 2011) | Larvae | Spawning occurs from March through July peaking in May and June. Embryos biome gratula within 64 hours, and two weeks later are articulate larvae. Larvae spend 3-4 weeks developing in the upper 20m of the water column. | | Muscat 1984; McCauley et al., 2012; Hamel and Mercier 2008; Rogers-Bennett and Ono, 2007 |
|  | Juveniles & Adults | Commonly found at shallower depths (30m) from December to July, before they disappear from shallow waters from August to November. | | Bruckner 2006; Conand 2006; Woodby et al., 2000; Hamel and Mercier, 2008. |
| *Mesocentrotus franciscanus*  Present from Kodiak Island, Alaska, US, to Baja California, Mexico (Kato et al., 1985) | Larvae | Northern California *M. franciscanus* populations spawn mostly in spring and summer. Southern California populations spawn in winter and spring. After the Embryo fertilizes, cleavage occurs within 25 hours, and larvae then spend 4-20 weeks (most commonly 7 weeks) in their pelagic phase. Satterthwaite found urchin larvae at 0-20m depth and up to 12km from shore. Other research on *M. Franciscanus* larvae found most larvae at <20m depth in very nearshore environments (<1 km). | | Kato and Schroeter, 1985;  Ebert and Russell, 1992; Davidson et al., 1998; Rogers-Bennett et al., 2007; Cameron and Schroeter 1980, Strathmann 1978, Miller and Emlet, 1997; Satterthwaite et al., 2021. |
|  | Juvenile | Larvae settle and develop at shallower depths than adults (10-20m) but also hide in spines of parents, suggesting many also settle to the same depth as abundant adult populations (50m). Northern California peak settlement is in mid-spring (May), while Southern California population peak settlement is later in spring and early summer. | | Rowley 1989; Ebert et al., 1994; Nishizaki and Ackerman, 2007 |
|  | Adult | Usually occupy shallow waters from mid-low intertidal zone to subtidal depths of 50m and are found as deep as 240m | | Kato and Schroeter, 1985; Carey Jr. and McCauley 1966; Lowe and Galloway 2020 |
| *Metacarnicus magister*  Present from Alaska, US through Point Conception, California, US (Woodby et al., 2005). | Embryo | Embryos extrude from September to November. After females extrude Embryos they migrate to shallow waters, dense aggregates at <20m. | | Orcutt et al., 1975; Hankin et al., 2001; Rasmuson 2013 |
|  | Early Zoea | Larvae released between November and February, and develop over 80-125 days. They occupy the upper 20-25m of the water column. Early stage zoea typically remain within 10km of shore, and in the upper 20m of the water column. | | Shanks et al., 2009; Grantham et al., 2003; Hankin et al., 2001; Rasmuson 2013; Morgan et al., 2014; Morgan et al., 2018 |
|  | Late Zoea | Larvae released between November and February, and develop over 80-125 days. Late-stage zoea aggregate at 20-60km from the coast at 0-40m depth, and occasionally travel up to 500km offshore | | Shanks et al., 2009; Grantham et al., 2003; Hankin et al., 2001; Rasmuson 2013; Morgan et al., 2014; Morgan et al., 2018 |
|  | Planktonic Megalopae | Pelagic post-larvae migrate to 0-70m depth and congregate at 30-100km offshore. | | Sulkin and McKeen 1996; Morgan et al., 2014; Reilly 1983; Shanks et al., 2009; Morgan et al. 2018 |
|  | Benthic Megalopae  & Juveniles | To avoid adults engaging in cannibalism, Juveniles crabs tend to remain in shallower waters (<55m) | | Sulkin and McKeen 1996; Hankin et al., 2001; Rasmuson 2013 |
|  | Adults | Most adults are caught at 55-85m, and are most commonly found between 30-90m, but can be found as deep as 220m. After females extrude Embryos they migrate to shallow waters forming dense aggregates at <20m. | | Hankin et al., 2001; Armstrong et al., 1989, Stone and O’Clair 2002, Rasmuson 2013. |
| *Pandalus jordani*  Present from Southeast Alaska, US, through Baja California, Mexico (Hannah and Jones, 2007). | Embryos | In northern California females carry Embryos from October-May, and in southern California from October-June. Peak hatching occurs in late March and early April. | | Dahlstrom 1961, 1970, 1973 |
|  | Early Larvae | Larvae spend 2-4 months in the pelagic larval phase and pass through 11-13 larval stages. Each larval stage lasts from 4-12 days, with an average of 7 days. The first two larval stages (early larvae) are found for a few weeks on either side of peak hatching, in <10m of water, and within 55km of shore. | | Modin and Cox 1967;  Pearcy 1970; Rothisberg and Miller 1983; Hannah et al., 2011; Frimodig et al., 2007; Bergstrom 2000 |
|  | Late Larvae | Larvae spend 2-4 months in the pelagic larval phase. At this time they inhabit down to 150m, and can reach up to 110km from shore. | | Pearcy 1970; Rothisberg and Miller 1983; Hannah et al., 2011; Frimodig et al., 2007; Bergstrom 2000 |
|  | Juveniles | Juveniles settle in late spring and summer at the shallower depths of the adult population (0-200m), and remain inshore for the first 2 years of life before migrating deeper. The highest concentration of Juveniles is found at (80-230m). | | Hannah et al., 2011; Frimodig et al., 2007; |
|  | Adults | Adults exist at depths of 0-1400m. The highest concentrations are from 80-230m depth. Adults undergo daily vertical migrations, staying in deeper waters close to the benthos during the day, and migrating to shallower water at night. | | Bergstrom 2000; Hannah 2011 |
| *Panulirus interruptus*  Typically found south of Point Conception, US, through southernmost Baja, California, Mexico (Wilson 1948). In recent years, found as far north as Monterey and Bodega Bay, US (Sanford et al., 2019) | Embryos | Fertilization occurs from January through May, peaking between February and April. Peak hatching in July-August when lobsters occupy shallower water (15-30m) | | Bodkin and Browne 1992  CDFW 2016 |
|  | Zoea | Spend 7-8 months as pelagic larvae. Larvae are found as deep as 137m and up to 530km from shore. However, it seems that most larvae aggregate within 120km from shore in Southern California and within 350km of shore in Baja California | | Engle 1979, Neilson 2011, Koslow et al., 2012; Funez-Rodriguez et al., 2015 |
|  | Benthic Megalopae | Post-larvae settle into shallow surfgrass and red algae in mid June to mid September at depths of <60m. They are considered postlarvae for 2-3 months | | CDFW 2016, Parker 1972 |
|  | Juveniles | After their post-larval phase they transition into Juveniles for 2-3 years. During this time they stay at <75m depth. | | Robles 1987, Neilson 2011 |
|  | Adults | Sub-adults and adults are found from 0-75m. Though many lobsters migrate to deep waters in winter (>50m) to avoid stormy winter conditions, returning to the shallows (15 - 30m) for spring and summer, Withy-Allen and Hovel (2013) did not find evidence of seasonal migrations in this species. | | Neilson 2011 |
| *Strongylocentrotus purpurpatus*  Present from Torch Bay, Alaska, US to Isla Cedros, Baja California, Mexico (Ebert et al., 2014) | Larvae | Spawning begins in late fall, and peaks in winter months. After the Embryo fertilizes, cleavage occurs within 72 hours. Larvae spend 4-20 weeks developing in the upper 0-20m of the water column within 12km from shore. | | Pearse et al., 1971; 1982; 1986; Conor 1972; Smith et al., 2008; Strathman 1978; 1987; Grantham et al., 2003; Satterthwaite et al., 2021. |
|  | Juvenile | Larvae settle and develop at shallower depths than adults (10-20m). Northern California settlement peaks twice, in March and July, while Southern California settlement peaks from April to May. | | Rowley 1989; Okamoto et al., 2020; |
|  | Adult | Usually occupy shallow waters from mid-low intertidal zone to subtidal depths of 50m and are found as deep as 160m | | Kato and Schroeter, 1985; Morris et al., 1980; Light and Smith. 2007. |

Table 2. Predicted effects of exposure to acidified conditions. Each effect is assigned a consequence score (C_i_), reflective of no (1), sublethal (2) or lethal (3) anticipated effects; and an uncertainty score (Uc_i_) reflecting low (1), moderate (2) or high (3) uncertainty, gleaned from how the number of studies, agreement among studies, and degree of relatedness between species used in laboratory studies and species of interest for this study.

| **Org.** | **Life Stage** | **Effects** | **Citations** | **C_i_** | **Uc_i_** |
| --- | --- | --- | --- | --- | --- |
| *A. parvimensus* | Larvae | Growth rate (no effect), minimal impact on development | Yuan et al., 2015 | 2 | 3 |
|  | Juveniles | Assumed same as adult | N/A | 2 | 3 |
|  | Adults | Growth | Yuan et al., 2016; Shi et al., 2021 | 2 | 3 |
| *M. franciscanus* | Larvae | Slightly reduced growth; however; strong response to selection suggesting organisms could rapidly adapt to changing conditions | Sunday et al., 2011 | 1.5 | 2 |
|  | Juveniles | Respiration, grazing, growth, net calcification; however; results come from experiments with concurrent low pH and low DO (upwelling conditions) | Donham et al., 2022; 2023 | 2 | 3 |
|  | Adults | Assumed same as juveniles | N/A | 1.5 | 3 |
| *M. magister* | Embryos | Delayed hatching | Miller et al., 2016 | 2 | 2 |
|  | Larvae | Reduced survival, movement, prolonged development rate | Christmas, 2013; Descoteaux, 2014; Miller et al., 2016 | 3 | 1 |
|  |  |  |  |  |  |
|  | Megalopae | Damage to mechanoreceptor; slowed growth | Bednaršek et al. 2020;  McElhany et al., 2022 | 2 | 2 |
|  |  |  |  |  |  |
|  | Juveniles | No effect; assumed same as adults | N/A | 1.5 | 3 |
|  | Adults | No effect seen by Pane & Barry & Hans et al., altered foraging behaviour was seen by Durant et al., | Pane & Barry, 2007; Hans et al., 2014; Durant et al., 2023 | 1.5 | 2 |
| *P. jordani* | Embryos | No effect on hatching | Arnberg et al. 2012 | 1 | 2.5 |
|  | Larvae | Increased development rate | Bechmann et al. 2011, Arnberg et al. 2012 | 2 | 2 |
|  | Juveniles | No effect; assumed same as adults | N/A | 1 | 3 |
|  | Adults | No effect | Hammer and Pedersen 2013 | 1 | 2.5 |
| *P. interruptus* | Embryos | No studies on closely related species | N/A | N/A | N/A |
|  | Larvae | No effect | Lowder 2019 | 1 | 2.5 |
|  | Juveniles | Changes to exoskeletal defenses | Lowder et al., 2022 | 2 | 2.5 |
|  | Adults | No effect | Knapp et al., 2016 | 1 | 3 |
| *S. purpurpatus* | Larvae | Reduced growth, delayed development. However, some studies show no effect of low pH. Others found high genetic variability and differential upregulation of genes at sites along pH mosaic, suggesting some populations are more resilient to future conditions. | Stump et al., 2011; Yu et al., 2011; Matson et al., 2012; Kelly et al., 2013; Padilla-Gamiño et al., 2013; Pespeni et al., 2013a,b; Evans et al., 2013; 2017; Garrett et al., 2020 | 1.5 | 2 |
|  | Juvenile | Assumed same as adults | N/A | 2 | 3 |
|  | Adults | Coleomic fluid pH  Feeding  Growth Rate | Spicer et al., 2011; Taylor et al., 2014; Zhan et al., 2020 | 2 | 2 |

Table 3. Predicted effects of exposure to deoxygenation. Each effect is assigned a consequence score (C_i_), reflective of no (1), sublethal (2) or lethal (3) anticipated effects; and an uncertainty score (Uc_i_) reflecting low (1), moderate (2) or high (3) uncertainty, gleaned from how the number of studies, agreement among studies, and degree of relatedness between species used in laboratory studies and species of interest for this study.

| **Org.** | **Life Stage** | **Effects** | **Citations** | **C_i_** | **Uc_i_** |
| --- | --- | --- | --- | --- | --- |
| *A. Parvimensus* | Larvae | Settlement | Yu et al., 2021 | 2 | 3 |
|  | Juveniles | Assumed the same as the adult | N/A | 2 | 3 |
|  | Adults | Altered enzyme activity;  Degradation of respiratory trees | Huo et al., 2018; Li et al., 2019 | 2 | 3 |
| *M. franciscanus* | Larvae | No effect on survivorship | Eerkes-Medrano et al., 2013 | 1 | 3 |
|  | Juveniles | Respiration, grazing, growth, net calcification; however; results come from experiments with concurrent low pH and low DO (upwelling conditions) | Donham et al., 2022; 2023 | 2 | 3 |
|  | Adults | Reduced grazing rate | Low and Micheli, 2018 | 2 | 2 |
| *M. magiste*r | Embryos | Decreased respiration rate; delayed hatching | Fernandez et al., 2003 | 2 | 3 |
|  | Larvae | Decreased survival | Vargo & Sastry, 1977; Miller et al., 2002 | 3 | 3 |
|  |  |  |  |  |  |
|  | Megalopae | Decreased respiration | Gossner, 2018 | 2 | 2 |
|  |  |  |  |  |  |
|  | Juveniles | Decreased survival | Bancroft 2015 | 3 | 2 |
|  | Adults | Survival | Grantham et al., 2004; Barth et al., 2018 | 3 | 1 |
| *P. jordani* | Embryos | Decreased yolk size; Delayed hatching | Brouwer et al., 2007; Peruzza et al., 2018 | 2 | 3 |
|  | Larvae | Survival | Kang and Matsuda 1994; Miller et al. 2002 | 3 | 3 |
|  | Juveniles | Avoidant behaviour, Decreased growth, Observed Pcrit | Renaud 1986; Seidman and Lawrence 1985; Rosas et al. 1997 | 2 | 3 |
|  | Adults | Accelerated molt cycle, decreased feeding, excretion & predation rates | Peruzza et al. 2018; Sandberg et al. 1996 | 2 | 3 |
| *P. interruptus* | Embryos | No studies on closely related species | N/A | N/A | N/A |
|  | Larvae | Inactivity, Q02 reduction | Belman and Childress 1973;  Winget 1969 | 2 | 1.5 |
|  | Juveniles | N/A | Assumed same as adults | 2 | 3 |
|  | Adults | PCrit | Ocampo et al., 2003 | 2 | 2 |
| *S. Purpurpatus* | Larvae | No effect on survivorship | Eerkes-Medrano et al., 2013 | 1 | 3 |
|  | Juveniles | Respiration, grazing, growth, net calcification; however; results come from experiments with concurrent low pH and low DO (upwelling conditions) | Donham et al., 2022; 2023 | 2 | 3 |
|  | Adults | Reduced grazing rate | Low and Micheli, 2018 | 2 | 2 |

Table 4. Uncertainty in exposure from literature, which can be introduced by contradictory information, or lack of information on biogeographic and depth distribution, and for larval stages the seasonality and distance traveled offshore. Each category scored low (1), medium (2), or high (3) uncertainty. **P. interruptus* Biogeography set to 2 for all life stages to reflect ongoing poleward expansion. Life stages are abbreviated, embryos are “emb.”, larvae are “larv.”, and juveniles are “juv.”.

| **Species** | **Life Stage** | **Biogeo. Distrib.** | **Depth Distrib.** | **Distance offshore** | **Season.** | **# Sources** | **Lit. Consen-**  **sus** | **Final** | **Notes** |
| --- | --- | --- | --- | --- | --- | --- | --- | --- | --- |
| *A. parvimensis* | Larv. | 1 | 1 | 1 | 1 | 1 | 1 | 1 | N/A |
|  | Juv. | 1 | 3 | N/A | N/A | 2 | 2 | 2.5 | Few studies and little consensus on depth distribution |
|  | Adult | 1 | 3 | N/A | N/A | 1 | 2 | 2 | Lack of consensus on depth distribution |
| *M. francis-*  *canus* | Larv. | 1 | 1 | 1 | 1 | 1 | 1 | 1 | N/A |
|  | Juv. | 1 | 2 | N/A | N/A | 2 | 2 | 2 | Few studies and little consensus on depth distribution |
|  | Adult | 1 | 2 | N/A | N/A | 1 | 2 | 1.5 | Lack of consensus on depth distribution |
| *M. magister* | Emb. | 1 | 1 | N/A | 1 | 1 | 1 | 1 | N/A |
|  | Larv. | 1 | 1 | 1 | 2 | 1 | 1 | 1 | Seasonality shifts with latitude |
|  |  |  |  |  |  |  |  |  |  |
|  | Juv. | 1 | 2 | N/A | N/A | 2 | 1 | 1.5 | Depth distribution is inferred;  few sources |
|  |  |  |  |  |  |  |  |  |  |
|  | Adult | 1 | 2 | N/A | N/A | 1 | 2 | 1.5 | Lack of consensus on depth distribution |
| *P. jordani* | Emb. | 1 | 1 | N/A | 1 | 2 | 1 | 1 | Only 3 sources |
|  | Larv. | 1 | 1 | 1 | 1 | 1 | 1 | 1 | N/A |
|  | Juv. | 1 | 2 | N/A | N/A | 1 | 1 | 1 | Depth distribution somewhat unclear |
|  | Adult | 1 | 1 | N/A | N/A | 2 | 1 | 1 | Only two sources |
| *P. interrup-*  *tus* | Emb. | 2 | 1 | N/A | 2 | 3 | 1 | 2 | Peak hatching rather than full window; only one source |
|  | Larv. | 2 | 3 | 2 | 1 | 1 | 2 | 2 | Depth distribution is maxima; conflicting distance offshore |
|  | Juv. | 2 | 1 | N/A | N/A | 2 | 1 | 1.5 | Only two sources |
|  | Adult | 2 | 1 | N/A | N/A | 2 | 1 | 1.5 | N/A |
| *S. purpurpatus* | Larv. | 1 | 1 | 1 | 1 | 1 | 1 | 1 | N/A |
|  | Juv. | 1 | 2 | N/A | N/A | 2 | 2 | 2 | Few studies and little consensus on depth distribution |
|  | Adult | 1 | 2 | N/A | N/A | 1 | 2 | 1.5 | Lack of consensus on depth distribution |

Table 5. Number of months with average pH below the threshold of 7.6 (% exposure) and corresponding scaled exposure scores (E_i_)

| **Organism** | **Life Stage** | **% Exposure** | **Score (E_i_)** |
| --- | --- | --- | --- |
| *A. parvimensis* | Larvae | 3.6 | 1.1 |
|  | Juveniles | 7.0 | 1.2 |
|  | Adults | 7.0 | 1.2 |
| *M. franciscanus* | Larvae | 2.4 | 1.1 |
|  | Juveniles | 3.5 | 1.1 |
|  | Adults | 3.5 | 1.1 |
| *M. magister* | Embryos | 0 | 1.0 |
|  | Zoea | 1.9 | 1.1 |
|  | Megalopae | 4.8 | 1.1 |
|  | Juveniles | 5.8 | 1.2 |
|  | Adults | 8.4 | 1.2 |
| *P. jordani* | Embryos | 0 | 1.0 |
|  | Early Larvae | 3.8 | 1.1 |
|  | Late Larvae | 5.1 | 1.1 |
|  | Juveniles | 9.2 | 1.2 |
|  | Adults | 9.2 | 1.2 |
| *P. interruptus* | Embryos | 0 | 1.0 |
|  | Zoea | 1.9 | 1.1 |
|  | Megalopaeae | 0 | 1.0 |
|  | Juveniles | 0 | 1.0 |
|  | Adults | 0 | 1.0 |
| *S. purpurpatus* | Larvae | 0.8 | 1.0 |
|  | Juveniles | 3.5 | 1.1 |
|  | Adults | 3.5 | 1.1 |

Table 6. Percent of monthly averages falling below different pH threshold values (7.55, 7.6, 7.65), indicating exposure calculations’ sensitivity to selected thresholds

| **Organism** | **Life Stage** | **7.55** | **7.6** | **7.65** |
| --- | --- | --- | --- | --- |
| *A. parvimensis* | Larvae | 2.2 | 3.6 | 5.0 |
|  | Juveniles | 6.1 | 7.0 | 8.8 |
|  | Adults | 6.1 | 7.0 | 8.8 |
| *M. franciscanus* | Larvae | 1.2 | 2.4 | 3.1 |
|  | Juveniles | 2.5 | 3.5 | 4.8 |
|  | Adults | 2.5 | 3.5 | 4.8 |
| *M. magister* | Embryos | 0 | 0 | 0 |
|  | Zoea | 0 | 1.9 | 1.9 |
|  | Megalopae | 2.7 | 4.8 | 6.6 |
|  | Juveniles | 3.7 | 5.8 | 7.5 |
|  | Adults | 2.5 | 8.4 | 15.1 |
| *P. jordani* | Embryos | 0 | 0 | 21.8 |
|  | Early Larvae | 1.8 | 3.8 | 4.6 |
|  | Late Larvae | 3.3 | 5.1 | 5.9 |
|  | Juveniles | 4.3 | 9.2 | 25.7 |
|  | Adults | 4.3 | 9.2 | 25.7 |
| *P. interruptus* | Embryos | 0 | 0 | 0 |
|  | Zoea | 0.9 | 1.9 | 2.8 |
|  | Megalopaeae | 0 | 0 | 0 |
|  | Juveniles | 0 | 0 | 0 |
|  | Adults | 0 | 0 | 0 |
| *S. purpurpatus* | Larvae | 0.5 | 0.8 | 0.8 |
|  | Juveniles | 2.5 | 3.5 | 4.8 |
|  | Adults | 2.5 | 3.5 | 4.8 |

Table 7. Number of weeks with average dissolved oxygen below the threshold of 33% saturation (% exposure) and corresponding scaled exposure scores (E_i_).

| **Organism** | **Life Stage** | **% Exposure** | **Score (E_i_)** |
| --- | --- | --- | --- |
| *A. parvimensis* | Larvae | 0 | 1.0 |
|  | Juveniles | 0 | 1.0 |
|  | Adults | 0 | 1.0 |
| *M. franciscanus* | Larvae | 0.2 | 1.0 |
|  | Juveniles | 1.8 | 1.0 |
|  | Adults | 1.8 | 1.0 |
| *M. magister* | Embryos | 0 | 1.0 |
|  | Zoea | 0 | 1.0 |
|  | Megalopae | 2.9 | 1.1 |
|  | Juveniles | 3.9 | 1.1 |
|  | Adults | 19.6 | 1.5 |
| *P. jordani* | Embryo | 39.6 | 2.1 |
|  | Early Larvae | 0 | 1.0 |
|  | Late Larvae | 1.1 | 1.1 |
|  | Juveniles | 73.4 | 3.0 |
|  | Adults | 73.4 | 3.0 |
| *P. interruptus* | Embryo | 0 | 1.0 |
|  | Zoea | 0 | 1.0 |
|  | Megalopae | 0 | 1.0 |
|  | Juveniles | 0 | 1.0 |
|  | Adults | 0 | 1.0 |
| *S. purpurpatus* | Larvae | 0 | 1.0 |
|  | Juveniles | 1.1 | 1.0 |
|  | Adults | 1.1 | 1.0 |

Table 8. Percent of weekly averages falling below different dissolved oxygen saturation threshold values (24%,33%,39%), indicating exposure calculations’ sensitivity to selected thresholds

| **Organism** | **Life Stage** | **24%** | **33%** | **39%** |
| --- | --- | --- | --- | --- |
| *A. parvimensis* | Larvae | 0 | 0 | 0.1 |
|  | Juveniles | 0 | 0 | 0 |
|  | Adults | 0 | 0 | 0 |
| *M. franciscanus* | Larvae | 0 | 0.2 | 0.9 |
|  | Juveniles | 0.3 | 1.8 | 2.3 |
|  | Adults | 0.3 | 1.8 | 2.3 |
| *M. magister* | Embryos | 0 | 0 | 0 |
|  | Zoea | 0 | 0 | 0 |
|  | Megalopae | 0.9 | 2.9 | 4.8 |
|  | Juveniles | 1.4 | 3.9 | 6.4 |
|  | Adults | 6.5 | 19.6 | 34.8 |
| *P. jordani* | Embryos | 14.2 | 39.6 | 54.9 |
|  | Early Larvae | 0 | 0 | 0.1 |
|  | Late Larvae | 0 | 1.1 | 3.1 |
|  | Juveniles | 25.1 | 73.4 | 90.4 |
|  | Adults | 25.1 | 73.4 | 90.4 |
| *P. interruptus* | Embryos | 0 | 0 | 1.3 |
|  | Zoea | 0 | 0 | 0 |
|  | Megalopaeae | 0 | 0 | 0 |
|  | Juveniles | 0 | 0 | 0.2 |
|  | Adults | 0 | 0 | 0.2 |
| *S. purpurpatus* | Larvae | 0 | 0 | 0.1 |
|  | Juveniles | 0 | 1.1 | 1.5 |
|  | Adults | 0 | 1.1 | 1.5 |

Table 9. Adult life stage exposure to pH < 7.6 and dissolved oxygen < 33% saturation at common and full depth ranges.

|  |  |  | **pH % exposure** | | **DO % exposure** | |
| --- | --- | --- | --- | --- | --- | --- |
| **Species** | **Common Depth** | **Full Depth** | **Common** | **Full** | **Common** | **Full** |
| *A.parvimensis* | 0-60m | 0-60m | 7.0 | N/A | 0 | N/A |
| *M. franciscanus* | 0-50m | 0-284m | 3.5 | 6.5 | 1.8 | 49.5 |
| *M. magister* | 30-90m | 0-220m | 8.4 | 7.8 | 19.6 | 34.4 |
| *P. jordani* | 80-230m | 0-1400m | 9.2 | 26.3 | 73.4 | 92.5 |
| *P. interruptus* | 0-75m | 0-75m | 0 | N/A | 0 | N/A |
| *S. purpurpatus* | 0-50m | 0-160m | 3.5 | 5.1 | 1.1 | 8.1 |

Table 10. Percent exposures for each life stage along their northern (>37.7°N) and southern (<37.7°N) latitudinal ranges within California.

| **Species** | **Life Stage** | **Parameter** | **North (>37.7**°**N)** | **South (<37.7**°**N)** |
| --- | --- | --- | --- | --- |
| *M. franciscanus* | Larvae | pH | 1.1 | 3.8 |
|  |  | DO | 0.4 | 0 |
|  | Juveniles / Adults | pH | 1.3 | 4.6 |
|  |  | DO | 3.2 | 0 |
| *P. jordani* | Embryos | pH | 0 | 0 |
|  |  | DO | 0 | 37.5 |
|  | Larvae | pH | 3.9 | 5.0 |
|  |  | DO | 1.1 | 0 |
|  | Juveniles / Adults | pH | 9.0 | 10.3 |
|  |  | DO | 58.1 | 76 |
| *S. purpurpatus* | Larvae | pH | 1.3 | 3.8 |
|  |  | DO | 0 | 0 |
|  | Juveniles / Adults | pH | 1.3 | 4.6 |
|  |  | DO | 3.2 | 0 |

Table 11. Uncertainty in exposure from dissolved oxygen data coverage. Uncertainty score assesses coverage across latitude, depth, distance offshore, months, years, and number of total averages. Each category scored low (1), medium (2), or high (3) uncertainty. Final score rounded to nearest 0.5. Life stages are abbreviated, embryos are “emb.”, larvae are “larv.”, megalopae are “mglp”, and juveniles are “juv.”.

| **Species** | **Life Stage** | **Lat.** | **Depth** | **Offshore** | **Range of Months** | **Range of Years** | **# Weekly Avg** | **Final** | **Notes** |
| --- | --- | --- | --- | --- | --- | --- | --- | --- | --- |
| *A. parvimensis* | Larv. | 1 | 1 | 2 | 1 | 1 | 1 | 1 | Few data at >3km offshore |
|  | Juv. & Adults | 3 | 2 | N/A | 1 | 1 | 1 | 1.5 | No data north of 34.7°N; No data at > 21m |
| *M. francis-*  *canus* | Larv. | 1 | 1 | 2 | 1 | 1 | 1 | 1 | In Southern California few data > 3km offshore |
|  |  |  |  |  |  |  |  |  |  |
|  | Juv. & Adults | 2 | 1 | N/A | 1 | 1 | 1 | 1 | Large data gaps between 34.5-36°N and 38.5-41°N |
|  |  |  |  |  |  |  |  |  |  |
| *M. magister* | Emb. | 3 | 2 | N/A | 1 | 2 | 1 | 2 | Large latitudinal gaps from 38.5-41°N; <30 weekly averages at > 2.5m; Few data post-2018 |
|  | Early Zoea | 1 | 1 | 1 | 1 | 1 | 1 | 1 | N/A |
|  | Late Zoea | 3 | 1 | 2 | 1 | 2 | 1 | 1.5 | Few data outside 41°N; very few data at > 40km offshore; most data from 2016 |
|  | Planktonic Mglp. | 3 | 1 | 1 | 1 | 2 | 1 | 1.5 | Very few data outside 41°N; most data from 2016 |
|  | Benthic Mglp. | 2 | 2 | N/A | 1 | 1 | 1 | 1.5 | Large data gaps from 38.5-41°N; no data between 5 and 30m; |
|  | Juv. | 2 | 3 | N/A | 1 | 1 | 1 | 1.5 | Large data gaps from 38.5-41°N;~ no data from 5 - 30m, and very few data at > 30m |
|  | Adults | 2 | 3 | N/A | 1 | 1 | 1 | 1.5 | Large data gaps between 39-41°N; very few data at > 30m |
| *P. jordani* | Emb. | 3 | 3 | N/A | 1 | 1 | 1 | 2 | Only data from 41°N in Northern California, in Southern California observations only at 200 - 220m |
|  | Early Larv. | 2 | 1 | 2 | 1 | 2 | 1 | 1.5 | Overrepresentation of data from 41°N, few data post-2018 |
|  | Late Larv. | 2 | 1 | 2 | 1 | 2 | 1 | 1.5 | Few data outside 41N in Northern California (>37.7°N); few data > 10km offshore in south; Few data after 2018 |
|  | Juv. & Adults | 2 | 3 | N/A | 1 | 2 | 1 | 2 | Overrepresentation of data from 41°N;, over-representation of data at 200m; few data post-2018 |
| *P. interrup-*  *tus* | Emb. | 1 | 2 | N/A | 1 | 1 | 2 | 1.5 | Few observations at > 15m depth; few data overall |
|  | Zoea | 1 | 1 | 3 | 1 | 2 | 1 | 1.5 | No data > 60km from shore; few data post-2018 |
|  | B Mglp. | 1 | 2 | N/A | 1 | 2 | 1 | 1.5 | Few observations at > 15m depth |
|  | Juv. & Adults | 1 | 3 | N/A | 1 | 1 | 1 | 1.5 | Few observations at > 15m depth |
|  |  |  |  |  |  |  |  |  |  |
| *S. purpurpatus* | Larv. | 1 | 1 | 2 | 1 | 1 | 1 | 1 | Few data at >3km offshore |
|  | Juv. & Adults | 2 | 1 | N/A | 1 | 1 | 1 | 1 | Large data gaps between 34.5-36°N and 38.5-41°N |

Table 12. Uncertainty in exposure from pH data coverage. Uncertainty score assesses coverage across latitude, depth, distance offshore, months, years, and number of total averages. Each category scored low (1), medium (2), or high (3) uncertainty. Life stages are abbreviated, embryos are “emb.”, larvae are “larv.”, megalopae are “mglp”, and juveniles are “juv.”.

| **Species** | **Life Stage** | **Lat.** | **Depth** | **Offshore** | **Range of Months** | **Range of Years** | **#/Monthly averages** | **Final** | **Notes** |
| --- | --- | --- | --- | --- | --- | --- | --- | --- | --- |
| *A. parvimensis* | Larv. | 2 | 1 | 3 | 1 | 1 | 1 | 1.5 | Most data from 34.5°N; very few data from >3km offshore |
|  | Juv. & Adults | 3 | 2 | N/A | 1 | 1 | 1 | 1.5 | No data north of 34.7°N; No data at > 21m |
| *M. francis-*  *canus* | Larv. | 1 | 1 | 2 | 1 | 1 | 1 | 1 | Few observations > 3km offshore |
|  |  |  |  |  |  |  |  |  |  |
|  | Juv. & Adults | 1 | 2 | N/A | 1 | 1 | 1 | 1 | Overrepresent. data at < 15m, <10 data at > 30m |
|  |  |  |  |  |  |  |  |  |  |
| *M. magister* | Emb. | 1 | 2 | N/A | 1 | 2 | 2 | 1.5 | Very little data overall; large latitudinal gaps from 38.5-41°N; <10 data below 2m in monthly averages; Few data post-2018; <50 monthly averages |
|  | Early. Zoea | 1 | 2 | 1 | 1 | 1 | 1 | 1 | Most data from 38.5N |
|  |  |  |  |  |  |  |  |  |  |
|  | Late. Zoea | 3 | 1 | 2 | 1 | 2 | 2 | 2 | <100 monthly averages, Few data outside 41°N; very few data > 40km offshore; most data from 2016 |
|  | Pelagic. Mglp. | 3 | 1 | 1 | 1 | 2 | 2 | 2 | <100 monthly averages, few data outside 41°N; most data from 2016 |
|  | Benthic. Mglp. | 1 | 3 | N/A | 1 | 1 | 1 | 1.5 | Very few data  at > 15m |
|  | Juv. | 1 | 3 | N/A | 1 | 1 | 1 | 1.5 | Very few data  at > 5m |
|  | Adults | 3 | 3 | N/A | 1 | 2 | 2 | 2 | Latitudinal gaps from 38 - 41°N; Very few data  at > 15m; most data from 2016; very few data overall |
| *P. jordani* | Emb. | 3 | 2 | N/A | 3 | 3 | 3 | 3 | Only data from 41°N in Northern California (>37.7°N); <100 monthly averages; overrepresentation of data from 200m; all Southern California data taken at 200m in May 2016 |
|  | Early Larvae | 2 | 1 | 2 | 1 | 2 | 1 | 1.5 | Few data outside 41°N in North and few data > 10km offshore in Southern California; few data in recent years |
|  | Late Larvae | 1 | 1 | 2 | 1 | 1 | 1 | 1 | N/A |
|  | Juv. & Adults | 2 | 1 | N/A | 1 | 1 | 1 | 1 | Overrepresentation of data from 38.5° and 41°N |
| *P. interrup-*  *tus* | Emb. | 1 | 3 | N/A | 1 | 1 | 1 | 1.5 | Very few observations at  > 15m |
|  | Zoea | 1 | 1 | 3 | 1 | 1 | 1 | 1.5 | Most data from 34.5°N; <10 observations > 20km offshore |
|  | Benthic Mglp. | 1 | 2 | N/A | 1 | 1 | 2 | 1.5 | Very few observations at > 15m; <100 monthly averages |
|  | Juv & Adults | 1 | 3 | N/A | 1 | 1 | 1 | 1.5 | Very few observations at > 15m |
|  |  |  |  |  |  |  |  |  |  |
| *S. purpuratus* | Larv. | 1 | 1 | 2 | 1 | 1 | 1 | 1 | Few data from >3km offshore |
|  | Juv. & Adults | 1 | 2 | N/A | 1 | 1 | 1 | 1 | Overrepresent. data at < 15m, <10 weekly averages at > 30m |

Table 13. Exposure, consequence, and vulnerability scores for each life stage, and species range in vulnerability for pH < 7.6.

| **Species** | **Life Stage** | **Exposure (Ei)** | **Consequence (Ci)** | **Vulnerability (Vi)** | **Range in Vi (Rv)** |
| --- | --- | --- | --- | --- | --- |
| *A. parvimensis* | Larvae | 1.1 | 1.5 | 1.7 | 0.7 |
|  | Juveniles | 1.2 | 2 | 2.4 |  |
|  | Adults | 1.2 | 2 | 2.4 |  |
| *M. franciscanus* | Larvae | 1.1 | 1.5 | 1.7 | 0.5 |
|  | Juveniles | 1.1 | 2 | 2.2 |  |
|  | Adults | 1.1 | 2 | 2.2 |  |
| *M. magister* | Embryos | 1.0 | 2 | 2 | 1.5 |
|  | Zoea | 1.1 | 3 | 3.3 |  |
|  | Megalopae | 1.1 | 2 | 2.2 |  |
|  | Juveniles | 1.2 | 1.5 | 1.8 |  |
|  | Adults | 1.2 | 1.5 | 1.8 |  |
| *P. jordani* | Embryos | 1.0 | 1 | 1 | 1.1 |
|  | Early Larvae | 1.1 | 2 | 2.1 |  |
|  | Late Larvae | 1.1 | 2 | 2.1 |  |
|  | Juveniles | 1.2 | 1 | 1.2 |  |
|  | Adults | 1.2 | 1 | 1.2 |  |
| *P. interruptus* | Embryos | 1.0 | N/A | N/A | 0 |
|  | Zoea | 1.1 | 1 | 1 |  |
|  | Megalopae | 1.0 | 1 | 1 |  |
|  | Juveniles | 1.0 | 1 | 1 |  |
|  | Adults | 1.0 | 1 | 1 |  |
| *S. purpurpatus* | Larvae | 1.0 | 1.5 | 1.5 | 0.7 |
|  | Juveniles | 1.1 | 2 | 2.2 |  |
|  | Adults | 1.1 | 2 | 2.2 |  |

Table 14. Exposure, consequence, and vulnerability scores for each life stage, and species range in vulnerability for dissolved oxygen < 33% saturation.

| **Species** | **Life Stage** | **Exposure (Ei)** | **Consequence (Ci)** | **Vulnerability (Vi)** | **Range in Vi (Rv)** |
| --- | --- | --- | --- | --- | --- |
| *A. parvimensis* | Larvae | 1.0 | 2 | 2 | 0 |
|  | Juveniles | 1.0 | 2 | 2 |  |
|  | Adults | 1.0 | 2 | 2 |  |
| *M. franciscanus* | Larvae | 1.0 | 1 | 1 | 1 |
|  | Juveniles | 1.0 | 2 | 2 |  |
|  | Adults | 1.0 | 2 | 2 |  |
| *M. magister* | Embryos | 1.0 | 2 | 2 | 3.1 |
|  | Zoea | 1.0 | 3 | 3 |  |
|  | Megalopae | 1.1 | 2 | 2.2 |  |
|  | Juveniles | 1.1 | 3 | 3.3 |  |
|  | Adults | 1.5 | 3 | 4.5 |  |
| *P. jordani* | Embryos | 2.1 | 2 | 4.2 | 3.4 |
|  | Early Larvae | 1.0 | 3 | 3 |  |
|  | Late Larvae | 1.1 | 3 | 3 |  |
|  | Juveniles | 3 | 2 | 6 |  |
|  | Adults | 3 | 2 | 6 |  |
| *P. interruptus* | Embryos | 1.0 | N/A | N/A | 1 |
|  | Zoea | 1.0 | 3 | 3 |  |
|  | Megalopae | 1.0 | 2 | 2 |  |
|  | Juveniles | 1.0 | 2 | 2 |  |
|  | Adults | 1.0 | 2 | 2 |  |
| *S. purpurpatus* | Larvae | 1.0 | 1 | 1 | 1 |
|  | Juveniles | 1.0 | 2 | 2 |  |
|  | Adults | 1.0 | 2 | 2 |  |

Table 15. Life stage uncertainty in dissolved oxygen consequence, exposure, and vulnerability. The final uncertainty in exposure scores are the average of uncertainty in exposure from literature and from data coverage.

| **Species** | **Life Stage** | **Uc** | **Ue from lit.** | **Ue from data** | **Ue Final** | **Uv** | **Range (Ru)** |
| --- | --- | --- | --- | --- | --- | --- | --- |
| *A. parvimensis* | Larvae | 3 | 1 | 1 | 1 | 3 | 2.25 |
|  | Juveniles | 3 | 2.5 | 1 | 1.75 | 5.25 |  |
|  | Adults | 3 | 2 | 1.5 | 1.75 | 5.25 |  |
| *M. franciscanus* | Larvae | 3 | 1 | 1 | 1 | 3 | 2 |
|  | Juveniles | 3 | 2 | 1 | 1.5 | 4.5 |  |
|  | Adults | 2 | 1.5 | 1 | 1.25 | 2.5 |  |
| *M. magister* | Embryos | 3 | 1 | 2 | 1.5 | 4.5 | 3 |
|  | Zoea | 3 | 1 | 1.25 | 1.1 | 3.3 |  |
|  | Megalopae | 2 | 1 | 1.5 | 1.5 | 3 |  |
|  | Juveniles | 2 | 1.5 | 1.5 | 1.5 | 3 |  |
|  | Adults | 1 | 1.5 | 1.5 | 1.5 | 1.5 |  |
| *P. jordani* | Embryo | 3 | 1 | 2 | 1.5 | 4.5 | 0.75 |
|  | Early Larvae | 3 | 1 | 1.5 | 1.25 | 3.75 |  |
|  | Late Larvae | 3 | 1 | 1.5 | 1.25 | 3.75 |  |
|  | Juveniles | 3 | 1 | 2 | 1.5 | 4.5 |  |
|  | Adults | 3 | 1 | 2 | 1.5 | 4.5 |  |
| *P. interruptus* | Embryos | N/A | 2 | 1.5 | 1.75 | N/A | 1.9 |
|  | Zoea | 1.5 | 2 | 1.5 | 1.75 | 2.625 |  |
|  | Megalopae | 1.5 | 2 | 1.5 | 1.75 | 2.625 |  |
|  | Juveniles | 3 | 1.5 | 1.5 | 1.5 | 4.5 |  |
|  | Adults | 2 | 1.5 | 1.5 | 1.5 | 3 |  |
| *S. purpurpatus* | Larvae | 3 | 1 | 1 | 1 | 3 | 2 |
|  | Juveniles | 3 | 2 | 1 | 1.5 | 4.5 |  |
|  | Adults | 2 | 1.5 | 1 | 1.25 | 2.5 |  |

Table 16. Uncertainty in pH consequence, exposure, and vulnerability. Final uncertainty in exposure scores are the average of uncertainty in exposure from literature and from data coverage.

| **Species** | **Life Stage** | **Uc** | **Ue from lit.** | **Ue from data** | **Ue Final** | **Uv** | **Range (Rv)** |
| --- | --- | --- | --- | --- | --- | --- | --- |
| *A. parvimensis* | Larvae | 3 | 1 | 1.5 | 1.25 | 3.75 | 2.25 |
|  | Juveniles | 3 | 2.5 | 1.5 | 2 | 6 |  |
|  | Adults | 3 | 2 | 1.5 | 1.75 | 5.25 |  |
| *M. franciscanus* | Larvae | 2 | 1 | 1 | 1 | 2 | 2.5 |
|  | Juveniles | 3 | 2 | 1 | 1.5 | 4.5 |  |
|  | Adults | 3 | 1.5 | 1 | 1.5 | 4.5 |  |
| *M. magister* | Embryos | 2 | 1 | 1.5 | 1.25 | 2.5 | 3.25 |
|  | Zoea | 1 | 1 | 1.5 | 1.25 | 1.25 |  |
|  | Megalopae | 2 | 1 | 1.75 | 1.5 | 3 |  |
|  | Juveniles | 3 | 1.5 | 1.5 | 1.5 | 4.5 |  |
|  | Adults | 2 | 1.5 | 2 | 1.75 | 3.5 |  |
| *P. jordani* | Embryo | 2.5 | 1 | 3 | 2 | 4.5 | 2.5 |
|  | Early Larvae | 2 | 1 | 1.5 | 1.25 | 2.5 |  |
|  | Late Larvae | 2 | 1 | 1 | 1 | 2 |  |
|  | Juveniles | 3 | 1 | 1 | 1 | 3 |  |
|  | Adults | 2.5 | 1 | 1 | 1 | 2.5 |  |
| *P. interruptus* | Embryos | N/A | 2 | 1.5 | 1.75 | N/A | 0.75 |
|  | Zoea | 2.5 | 2 | 1.5 | 1.75 | 4.4 |  |
|  | Megalopaeae | 2.5 | 2 | 1.5 | 1.75 | 4.4 |  |
|  | Juveniles | 2.5 | 1.5 | 1.5 | 1.5 | 3.75 |  |
|  | Adults | 3 | 1.5 | 1.5 | 1.5 | 4.5 |  |
| *S. purpurpatus* | Larvae | 2 | 1 | 1 | 1 | 2 | 2.5 |
|  | Juveniles | 3 | 2 | 1 | 1.5 | 4.5 |  |
|  | Adults | 2 | 1.5 | 1 | 1.25 | 2.5 |  |

**S1 References**

Arnberg M, Calosi P, Spicer J, Tandberg A, Nilsen M, Westerlund S et al. Elevated temperature elicits greater effects than decreased pH on the development, feeding and metabolism of northern shrimp (Pandalus borealis) larvae. Marine Biology. 2012:1- 12.

Bechmann, RK, Taban IC, Westerlund S, Godal BF, Arnberg M, Vingen S, et al. Effects of ocean acidification on early life stages of shrimp (Pandalus borealis) and mussel (Mytilus edulis). J Toxicol Environ Health A. 2011. 74:424-438.

Bednaršek N, Feely RA, Beck MW, Alin SR, Siedlecki SA, Calosi P, et al. Exoskeleton dissolution with mechanoreceptor damage in larval Dungeness crab related to severity of present-day ocean acidification vertical gradients. Science of The Total Environment. 2020 May 10;716:136610.

Bergström BI. The biology of Pandalus.

Bodkin JL, Browne L. Molt frequency and size-class distribution in the California spiny lobster (Panulirus interruptus) as indicated by beach-cast carapaces at San Nicolas Island, California. California Fish and Game. 1992. 78: 136-144.

Bruckner A. Management and conservation strategies and practices for sea cucumbers. In workshop on the conservation of sea cucumbers in the families Holothuriidae and Stichopodidae 2006 Aug (p. 74).

Carey Jr AG, McCauley JE. RESEARCH IN PROGRESS. NEE'TO/V. 1966 Jul:119.

California Department of Fish and Wildlife (CFDW). California Spiny Lobster Fishery Management Plan. 2016. 239 p

Cameron RA, Schroeter SC. Sea urchin recruitment: effect of substrate selection on juvenile distribution. Mar. Ecol. Prog. Ser. 1980 Jan 1;2(3):243-7.

Carlton JT, editor. The Light and Smith manual: intertidal invertebrates from central California to Oregon. Univ of California Press; 2023 Sep 1.

Chávez EA, Salgado-Rogel ML, Palleiro-Nayar J. Stock Assessment of the warty sea cucumber fishery (Parastichopus parvimensis) of NW Baja California. California Cooperative Oceanic Fisheries Investigations Reports. 2011 Nov 30;52:136-47.

Christmas, AMF. Effects of ocean acidification on dispersal behavior in the larval stage of the Dungeness crab and the Pacific Green Shore crab. Western Washington University. 2013.

Conand C. Sea cucumber biology, taxonomy, distribution and conservation status. Inworkshop on the conservation of sea cucumbers in the families Holothuriidae and Stichopodidae 2006 (Vol. 33).

Conor JJ. Gonad growth in the sea urchin, Strongylocentrotus purpuratus (Stimpson)(Echinodermata: Echinoidea) and the assumptions of gonad index methods. Journal of Experimental Marine Biology and Ecology. 1972 Nov 1;10(2):89-103.

Dahlstrom WA. The California ocean shrimp fishery. Pac. Mar. Fish. Comm. Bull. 1961;5:17-23.

Dahlstrom, WA. Synopsis of biological data on the ocean shrimp Pandalus jordani Rathbun. FAO Fisheries Report 1970; 57:377-1416.

Dahlstrom, WA. Status of the California ocean shrimp resource and its management. Marine Fisheries Review. 1973; 35:55-59.

Davidson EH, Cameron RA, Ransick A. Specification of cell fate in the sea urchin embryo: summary and some proposed mechanisms. Development. 1998 Sep 1;125(17):3269-90.

Descoteaux, R. Effects of ocean acidification on development of Alaskan crab larvae. University of Alaska Fairbanks. 2014.

Donham EM, Strope LT, Hamilton SL, Kroeker KJ. Coupled changes in pH, temperature, and dissolved oxygen impact the physiology and ecology of herbivorous kelp forest grazers. Global Change Biology. 2022;28(9):3023–39.

Donham EM, Flores I, Hooper A, O’Brien E, Vylet K, Takeshita Y, et al. Population-specific vulnerability to ocean change in a multistressor environment. Science Advances. 2023 Jan 20;9(3):eade2365.

Durant A, Khodikian E, Porteus CS. Ocean acidification alters foraging behaviour in Dungeness crab through impairment of the olfactory pathway. Global Change Biology. 2023;29(14):4126–39.

Ebert TA, Russell MP. Growth and mortality estimates for red sea urchin Strongylocentrotus franciscanus from San Nicolas Island, California. Marine ecology progress series. Oldendorf. 1992 Mar 1;81(1):31-41.

Ebert TA, Schroeter SC, Dixon JD, Kalvass P. Settlement patterns of red and purple sea urchins (Strongylocentrotus franciscanus and S. purpuratus) in California, USA. Marine Ecology Progress Series. 1994 Aug 11:41-52.

Ebert TA, Hernández JC, Clemente S. Annual reversible plasticity of feeding structures: cyclical changes of jaw allometry in a sea urchin. Proceedings of the Royal Society B: Biological Sciences. 2014 Mar 22;281(1779):20132284.

Engle JM. Ecology and growth of juvenile California spiny lobster, Panulirus

interruptus (Randall) [Doctoral dissertation]. University of Southern California. 1979; 298 p.

Frimodig A, Horeczko M, Mason T, Owens B, Prall M, Wertz S, Tillman T. Information concerning the pink shrimp trawl fishery off northern California.

Evans TG, Chan F, Menge BA, Hofmann GE. Transcriptomic responses to ocean acidification in larval sea urchins from a naturally variable pH environment. Molecular Ecology. 2013;22(6):1609–25.

Funes-Rodríguez R, Ruíz-Chavarría JA, González-Armas R, Durazo R, Guzmán-del Proó SA. Influence of hydrographic conditions on the distribution of spiny lobster larvae off the west coast of Baja California. Transactions of the American Fisheries Society. 2015 Nov 2;144(6):1192-205.

Garrett AD, Brennan RS, Steinhart AL, Pelletier AM, Pespeni MH. Unique Genomic and Phenotypic Responses to Extreme and Variable pH Conditions in Purple Urchin Larvae. Integrative and Comparative Biology. 2020 Aug 1;60(2):318–31.

Grantham BA, Eckert GL, Shanks AL. Dispersal Potential of Marine Invertebrates in Diverse Habitats. Ecological Applications. 2003;13(sp1):108–16.

Hamel JF, Mercier A. Population status, fisheries and trade of sea cucumbers in temperate areas of the Northern Hemisphere. Sea cucumbers. A global review of fisheries and trade. FAO Fisheries and Aquaculture Technical Paper. 2008;516(2008):257-91.

Hammer K, Pedersen S. Deep-water prawn Pandalus borealis displays a relatively high pH regulatory capacity in response to CO2-induced acidosis. Mar Ecol Prog Ser. 2013 Oct 31;492:139–51.

Hankin DG, Diamond N, Mohr MS, Ianelli J. Growth and reproductive dynamics of adult female Dungeness crabs (Cancer magister) in northern California. Journal du Conseil: ICES Journal of Marine Science. 1989; 46:94-108

Hannah, RW. Variation in the distribution of ocean shrimp (Pandalus jordani) recruits: links with coastal upwelling and climate change. Fisheries Oceanography. 2011; 20:305-313.

Hannah RW, Jones SA. Effectiveness of bycatch reduction devices (BRDs) in the ocean shrimp (Pandalus jordani) trawl fishery. Fisheries Research. 2007 Jun 1;85(1-2):217-25.

Hans S, Fehsenfeld S, Treberg J, Weihrauch D. Acid–base regulation in the Dungeness crab (Metacarcinus magister). Marine Biology. 2014;1-15.

Kato S, Schroeter SC. Biology of the Red Sea Urchin, Strongylocentrotus franciscanus, and Its Fishery in California. Marine Fisheries Review. 1985;20.

Kelly MW, Hofmann GE. Adaptation and the physiology of ocean acidification. Functional Ecology. 2013;27(4):980–90.

Knapp JL, Bridges CR, Krohn J, Hoffman LC, Auerswald L. The effects of hypercapnia on the West Coast rock lobster (Jasus lalandii) through acute exposure to decreased seawater pH — Physiological and biochemical responses. Journal of Experimental Marine Biology and Ecology. 2016 Mar 1;476:58–64.

Koslow JA, Rogers-Bennett L, Neilson DJ. 2012. A time series of California spiny lobster (Panulirus interruptus) phyllosoma from 1951-2008 links abundance to warm water oceanographic conditions in southern California. California Cooperative Oceanic Fisheries Investigations Report 53: 132-139.

Lowder KB. Integrity of crustacean predator defenses under ocean acidification and warming conditions. University of California, San Diego; 2019.

Lowder KB, deVries MS, Hattingh R, Day JMD, Andersson AJ, Zerofski PJ, et al. Exoskeletal predator defenses of juvenile California spiny lobsters (Panulirus interruptus) are affected by fluctuating ocean acidification-like conditions. Front Mar Sci [Internet]. 2022 Aug 4 [cited 2024 Oct 21];9. Available from: https://www.frontiersin.org/journals/marine-science/articles/10.3389/fmars.2022.909017/full

Lowe AT, Galloway AW. Urchin Searchin’: Red urchins and drift kelp found at 284 m in the mesophotic zone. Ciencias Marinas. 2020 Dec 18;46(4):283–96.

McCauley BS, Wright EP, Exner C, Kitazawa C, Hinman VF. Development of an embryonic skeletogenic mesenchyme lineage in a sea cucumber reveals the trajectory of change for the evolution of novel structures in echinoderms. EvoDevo. 2012 Aug 9;3(1):17.

McElhany P, Busch DS, Lawrence A, Maher M, Perez D, Reinhardt EM, et al. Higher survival but smaller size of juvenile Dungeness crab (Metacarcinus magister) in high CO2. Journal of Experimental Marine Biology and Ecology. 2022 Oct 1;555:151781.

Miller BA, Emlet RB. Influence of nearshore hydrodynamics on larval abundance and settlement of sea urchins Strongylocentrotus franciscanus and S. purpuratus in the Oregon upwelling zone. Marine Ecology Progress Series. 1997 Feb 27;148:83-94.

Miller JJ, Maher M, Bohaboy E, Friedman CS, McElhany P. Exposure to low pH reduces survival and delays development in early life stages of Dungeness crab (Cancer magister). Mar Biol. 2016 Apr 25;163(5):118.

Modin JC, Cox KW. Post-embryonic development of laboratory-reared ocean shrimp, Pandalus jordani Rathbun. Crustaceana. 1967 Sep 1:197-219.

Morris RH, Abbott DP, Haderlie EC. Intertidal Invertebrates of California. Stanford, CA: Stanford University Press. 1980.

Morgan SG. Behaviorally Mediated Larval Transport in Upwelling Systems. Advances in Oceanography. 2014 Jul 17;2014:e364214.

Morgan SG, Miller SH, Robart MJ, Largier JL. Nearshore Larval Retention and Cross-Shelf Migration of Benthic Crustaceans at an Upwelling Center. Front Mar Sci [Internet]. 2018 [cited 2021 Feb 27];5. Available from: https://www.frontiersin.org/articles/10.3389/fmars.2018.00161/full

Muscat AM. Population dynamics and the effect on the infauna of the deposit-feeding holothurian Parastichopus parvimensis (Clark). 1984.

Neilson DJ. Assessment of the California Spiny Lobster (Panulirus interruptus). California Department of Fish and Wildlife. 2011;138 p.

Nishizaki MT, Ackerman JD. Juvenile–adult associations in sea urchins (Strongylocentrotus franciscanus and S. droebachiensis): protection from predation and hydrodynamics in S. franciscanus. Marine Biology. 2007 Mar;151:135-45.

Okamoto DK, Schroeter SC, Reed DC. Effects of ocean climate on spatiotemporal variation in sea urchin settlement and recruitment. Limnology and Oceanography. 2020;65(9):2076–91.

Orcutt, HG. et al. 1975. Dungeness crab research program, report for the year 1975. Mar. Re- sources Admin. Rep. (75-12): 1-77.

Padilla-Gamiño JL, Kelly MW, Evans TG, Hofmann GE. Temperature and CO(2) additively regulate physiology, morphology and genomic responses of larval sea urchins, Strongylocentrotus purpuratus. Proc Biol Sci. 2013 May 22;280(1759):20130155.

Pane EF, Barry JP. Extracellular acid-base regulation during short-term hypercapnia is effective in a shallow-water crab, but ineffective in a deep-sea crab. Marine Ecology Progress Series. 2007;334:1-9

Parker KP. Recruitment and behavior of Puerulus larvae and juveniles of the

California spiny lobster, Panulirus interruptus (Randall). San Diego State College.1972.

Pearcy WG. Vertical migration of the ocean shrimp, Pandalus jordani: a feeding and dispersal mechanism.

Pearse JS, Pearse VB. Skeletal growth zones in echinoids. American Zoologist. 1975;15: 731–753.

Pearse JS, Pearse VB, Davis KK. Photoperiodic regulation of gametogenesis and growth in the sea urchin Strongylocentrotus purpuratus. Journal of Experimental Zoology. 1986 Jan;237(1):107-18.

Pearse JS, and Eernisse DJ. Photoperiodic regulation of gametogenesis and gonadal growth in the sea star Pisaster ochraceus. Marine Biology, 1982;67: 121–125.

Pespeni MH, Chan F, Menge BA, Palumbi SR. Signs of Adaptation to Local pH Conditions across an Environmental Mosaic in the California Current Ecosystem. Integrative and Comparative Biology. 2013 Nov 1;53(5):857–70.

Pespeni MH, Sanford E, Gaylord B, Hill TM, Hosfelt JD, Jaris HK, et al. Evolutionary change during experimental ocean acidification. Proc Natl Acad Sci U S A. 2013 Apr 23;110(17):6937–42.

Rasmuson, LK. The Biology, Ecology and Fishery of the Dungeness crab, Cancer magister. Advancements in Marine Biology. 2013;65:95-148.

Reilly, PN. Dynamics of Dungeness crab, Cancer magister, larvae off central and northern California. Life history, environment, and mariculture studies of Dungeness crab, Cancer magister, with emphasis on the central California® fishery resource. Calif. Dept Fish Game, Fish. Bull. 1983; 172:57-84

Robles C. Predator foraging characteristics and prey population structure on a sheltered shore. Ecology. 1987;68: 1502-1514.

Rogers-Bennett L. Chapter 19 The ecology of Strongylocentrotus franciscanus and Strongylocentrotus purpuratus. Developments in Aquaculture and Fisheries Science. 2007 Dec 31;37:393–425.

Rothlisberg PC, Miller CB. Factors affecting the distribution, abundance and survival of Pandalus jordani (Decapoda, Pandalidae) larvae off the Oregon coast. Fisheries Bulletin. 1983;81:455-472

Rowley RJ. Settlement and recruitment of sea urchins (Strongylocentrotus spp.) in a sea-urchin barren ground and a kelp bed: are populations regulated by settlement or post-settlement processes? Marine Biology. 1989 Mar;100:485-94.

Sanford E, Sones JL, García-Reyes M, Goddard JHR, Largier JL. Widespread shifts in the coastal biota of northern California during the 2014–2016 marine heatwaves. Sci Rep. 2019 Mar 12;9(1):4216.

Satterthwaite E, Ryan J, Harvey J, Morgan S. Invertebrate larval distributions influenced by adult habitat distribution, larval behavior, and hydrodynamics in the retentive upwelling shadow of Monterey Bay, California, USA. Mar Ecol Prog Ser. 2021 Mar 4;661:35–47.

Shanks AL. Pelagic Larval Duration and Dispersal Distance Revisited. The Biological Bulletin. 2009;Jun; 216(3):373–85.

Shi W, Li Y, Dong Y, Xin M, Zhang X, Xu Q. The effect of ocean acidification on the enzyme activity of Apostichopus japonicus. Fish & Shellfish Immunology. 2021 Jan 1;108:1–6.

Strathmann R. Length of pelagic period in echinoderms with feeding larvae from the Northeast Pacific. Journal of experimental marine biology and ecology. 1978 Jan 1;34(1):23-7.

Strathmann R. Length of pelagic period in echinoderms with feeding larvae from the Northeast Pacific. Journal of Experimental Marine Biology and Ecology. 1978 Jan 1;34(1):23–7.

Stumpp M, Wren J, Melzner F, Thorndyke MC, Dupont ST. CO2 induced seawater acidification impacts sea urchin larval development I: Elevated metabolic rates decrease scope for growth and induce developmental delay. Comparative Biochemistry and Physiology Part A: Molecular & Integrative Physiology. 2011 Nov 1;160(3):331–40.

Sunday JM, Crim RN, Harley CDG, Hart MW. Quantifying Rates of Evolutionary Adaptation in Response to Ocean Acidification. PLOS ONE. 2011 Aug 9;6(8):e22881.

Stone RP, O'Clair CE. Behavior of female Dungeness crabs, Cancer magister, in a glacial southeast Alaska estuary: homing, brooding-site fidelity, seasonal movements, and habitat use. Journal of Crustacean Biology. 2002 May;22(2):481-92.

Sulkin SD, Mojica E, McKeen GL. Elevated summer temperature effects on megalopal and early juvenile development in the Dungeness crab, Cancer magister. Canadian Journal of Fisheries and Aquatic Sciences. 1996 Sep 1;53(9):2076-9.

Wilson, RC. A review of the southern California spiny lobster fishery. Calif. Fish. Game. 1948;34(2):71-80

Woodby D, Smiley S, Larson R. Depth and habitat distribution of Parastichopus californicus near Sitka, Alaska. Alaska Fishery Research Bulletin. 2000;7:22-32.

Yu PC, Matson PG, Martz TR, Hofmann GE. The ocean acidification seascape and its relationship to the performance of calcifying marine invertebrates: Laboratory experiments on the development of urchin larvae framed by environmentally-relevant pCO2/pH. Journal of Experimental Marine Biology and Ecology. 2011 Apr 30;400(1):288–95.

Yuan X, Shao S, Dupont S, Meng L, Liu Y, Wang L. Impact of CO2-driven acidification on the development of the sea cucumber Apostichopus japonicus (Selenka) (Echinodermata: Holothuroidea). Marine Pollution Bulletin. 2015 Jun 15;95(1):195–9.

Yuan X, Shao S, Yang X, Yang D, Xu Q, Zong H, et al. Bioenergetic trade-offs in the sea cucumber Apostichopus japonicus (Echinodermata: Holothuroidea) in response to CO2-driven ocean acidification. Environ Sci Pollut Res. 2016 May 1;23(9):8453–61.

Zhan Y, Cui D, Xing D, Zhang J, Zhang W, Li Y, et al. CO2-driven ocean acidification repressed the growth of adult sea urchin Strongylocentrotus intermedius by impairing intestine function. Marine Pollution Bulletin. 2020 Apr 1;153:110944.
