# Supplementary figures and images for: Assessing benthic invertebrate vulnerability to ocean acidification and de-oxygenation in California: The importance of effective oceanographic monitoring networks
Source: PLoS One. 2025 Feb 18;20(2):e0317906. doi: 10.1371/journal.pone.0317906 (PMC11835291; doi:10.1371/journal.pone.0317906)

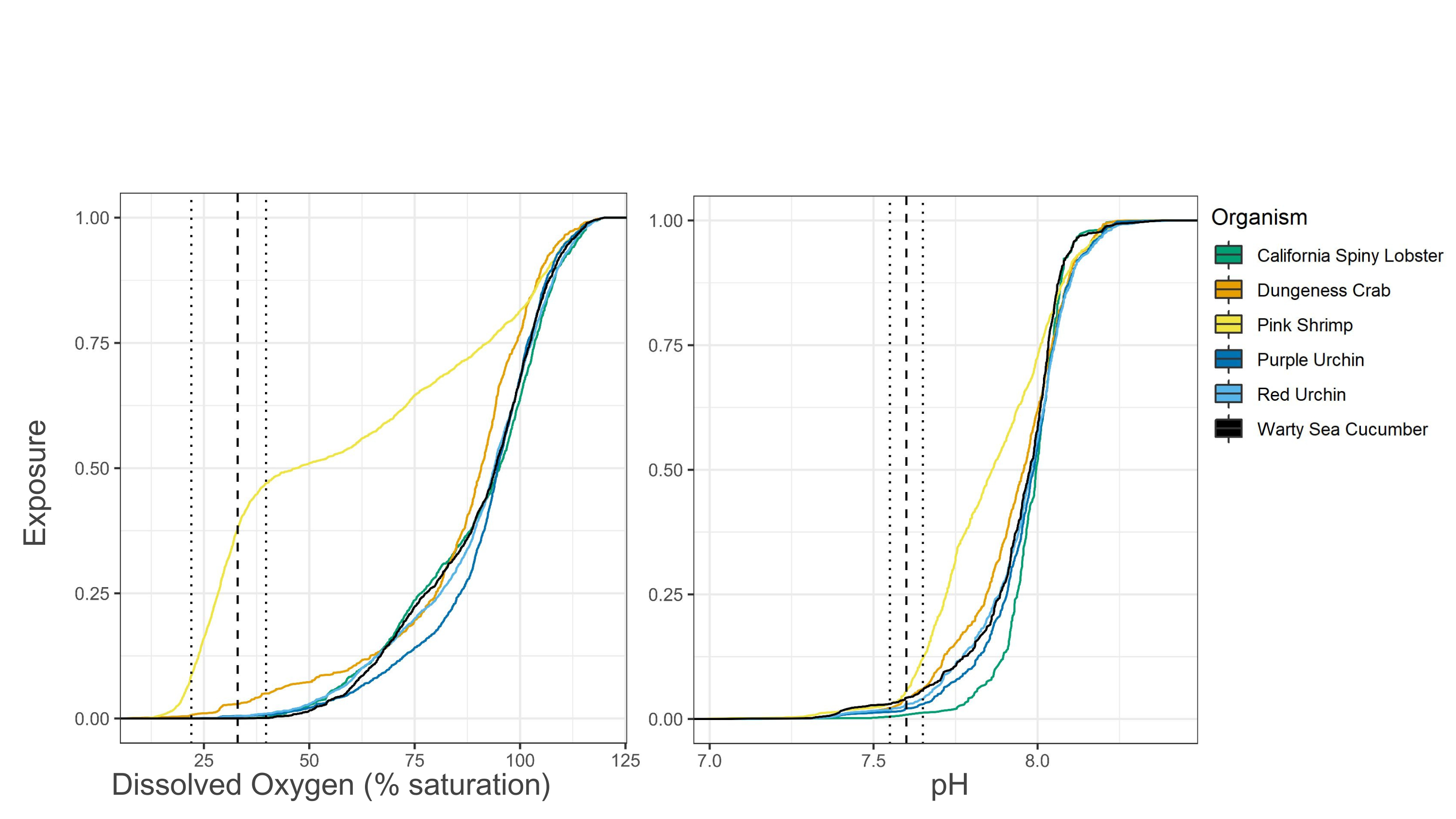

Supplement: S1 Fig — The dotted lines represent low and high pH and dissolved oxygen thresholds (7.55 and 7.65 for pH; 24% and 39% saturation for DO). The dashed line represents the thresholds used in this study (7.6 for pH, 33% saturation for DO). For pH, percent exposure is calculated as the number of monthly averages falling below the threshold. For dissolved oxygen, percent exposure is calculated as the number of weekly averages falling below the DO threshold. Exposures for P. jordani, C. magister increase significantly between the low and high thresholds. (PNG) [file pone.0317906.s003.png]

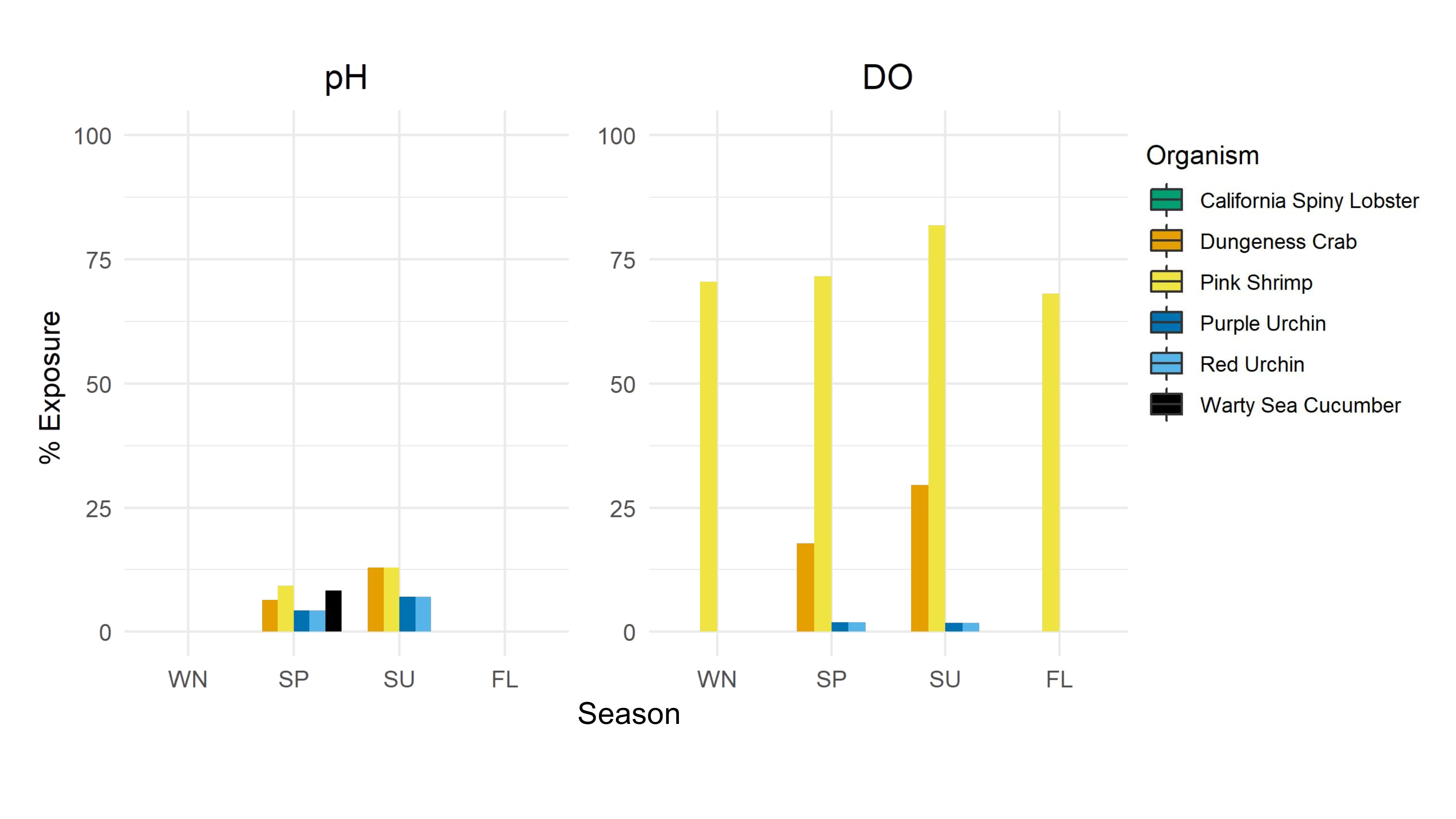

Supplement: S2 Fig — Winter includes data from January to March, spring includes data from April to June, summer includes data from July to September, and fall contains data from October to December. For P. interruptus, C. Magister, P. jordani, percent exposure is calculated as the number of monthly averages falling below the pH threshold. For M. franciscanus, S. purpurpatus, and A. parvimensis percent exposure is calculated as the number of weekly averages falling below the pH threshold. For all organisms percent exposure to dissolved oxygen is calculated using weekly averages. Exposures for P. jordani, and C. magister increase significantly between the mid and high thresholds. (PNG) [file pone.0317906.s004.png]

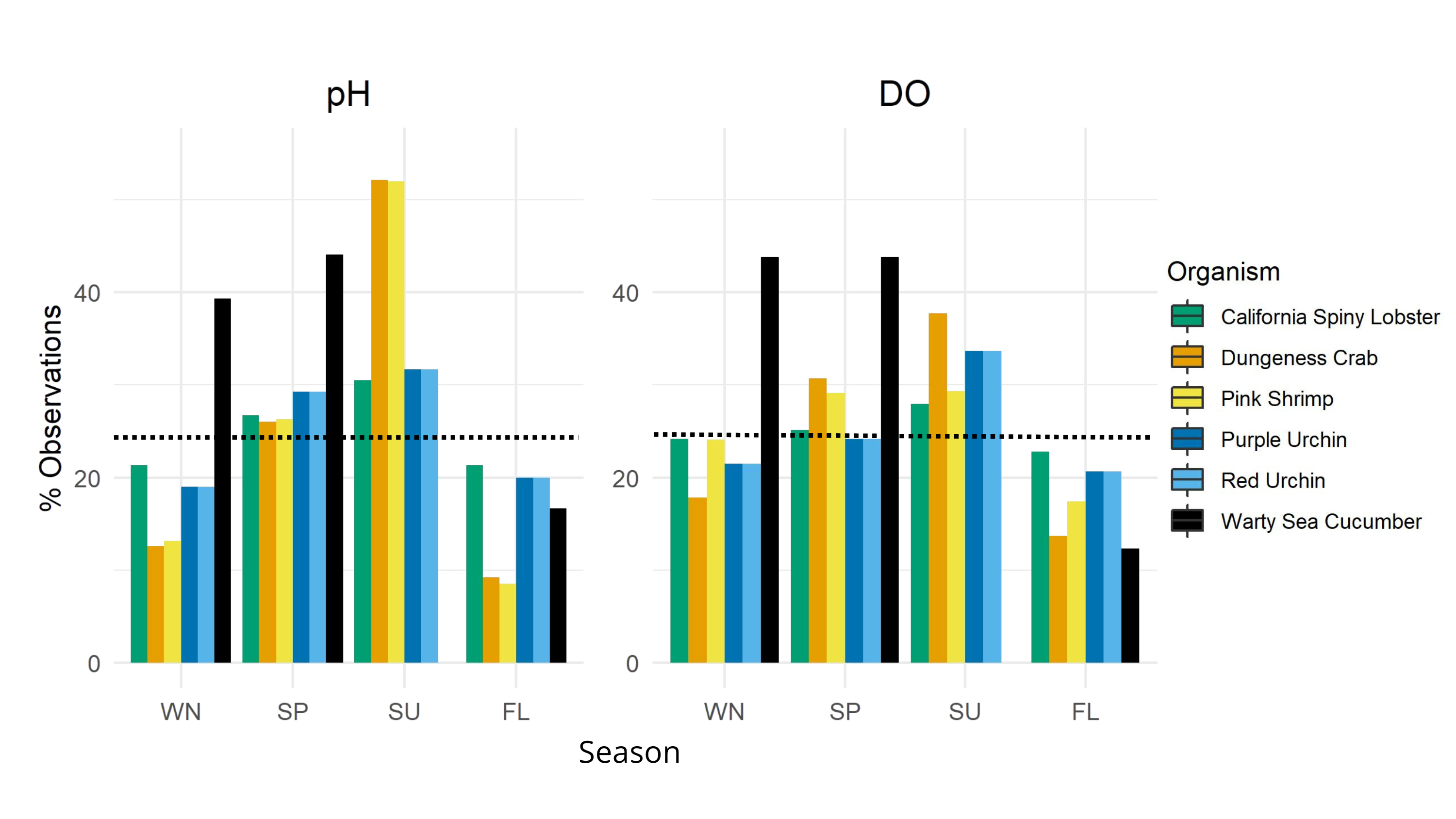

Supplement: S3 Fig — Winter includes data from January to March, spring includes data from April to June, summer includes data from July to September, and fall contains data from October to December. Spring and Summer pH observations are overrepresented, while Fall and Winter are underrepresented, with the exception of A. parvimensis (California warty sea cucumber), which moves from onshore to offshore in the summer months. Fall is underrepresented in dissolved oxygen observations, with a slight overrepresentation of other seasons. (PNG) [file pone.0317906.s005.png]
